# Gestational Diabetes Mellitus-Associated Hyperglycemia Impairs Glucose Transporter 3 Trafficking in Trophoblasts Through the Downregulation of AMP-Activated Protein Kinase

**DOI:** 10.3389/fcell.2021.722024

**Published:** 2021-11-02

**Authors:** Li Zhang, Xinyang Yu, Yue Wu, Huijia Fu, Ping Xu, Yangxi Zheng, Li Wen, Xiaotao Yang, Fumei Zhang, Mingyu Hu, Hao Wang, Xiyao Liu, Juan Qiao, Chuan Peng, Rufei Gao, Richard Saffery, Yong Fu, Hongbo Qi, Chao Tong, Mark D. Kilby, Philip N. Baker

**Affiliations:** ^1^Department of Obstetrics, The First Affiliated Hospital of Chongqing Medical University, Chongqing, China; ^2^State Key Laboratory of Maternal and Fetal Medicine of Chongqing Municipality, Chongqing, China; ^3^International Collaborative Laboratory of Reproduction and Development of the Chinese Ministry of Education, Chongqing Medical University, Chongqing, China; ^4^Department of Biochemistry and Molecular Biology, University of Texas McGovern Medical School, Houston, TX, United States; ^5^Department of Stem Cell Transplantation and Cell Therapy, MD Anderson Cancer Center, Houston, TX, United States; ^6^Laboratory of Reproductive Biology, School of Public Health and Management, Chongqing Medical University, Chongqing, China; ^7^Department of Paediatrics, Cancer, Disease and Developmental Epigenetics, Murdoch Children’s Research Institute, University of Melbourne, Parkville, VIC, Australia; ^8^Institute of Metabolism and Systems Research, University of Birmingham, Birmingham, United Kingdom; ^9^College of Life Sciences, University of Leicester, Leicester, United Kingdom

**Keywords:** AMP-activated protein kinase (AMPK), gestational diabetes mellitus (GDM), glucose transporter 3 (GLUT3), glucose uptake, trophoblast

## Abstract

AMP-activated protein kinase (AMPK) is an important regulator of glucose metabolism, and glucose transporter 3 (GLUT3) is an efficient glucose transporter in trophoblasts. Whether placental AMPK and GLUT3 respond accordingly to gestational diabetes mellitus (GDM) remains uncertain. Here, we explored the regulatory role of AMPK in the GLUT3-dependent uptake of glucose by placental trophoblasts and the viability of the cells. In this study, the level of glycolysis in normal and GDM-complicated placentas was assessed by LC-MS/MS. The trophoblast hyperglycemia model was induced by the incubation of HTR8/SVneo cells with a high glucose concentration. GDM animal models were generated with db/ + mice and C57BL/6J mice fed a high-fat diet, and AMPK was manipulated by the oral administration of metformin. The uptake of glucose by trophoblasts was assessed using 2-NBDG or 2-deoxy-D-[^3^H] glucose. The results showed that GDM is associated with impaired glycolysis, AMPK activity, GLUT3 expression in the plasma membrane (PM) and cell survival in the placenta. Hyperglycemia induced similar changes in trophoblasts, and these changes were rescued by AMPK activation. Both hyperglycemic db/ + and high-fat diet-induced GDM mice exhibited a compromised AMPK–GLUT3 axis and suppressed cell viability in the placenta as well as excessive fetal growth, and all of these effects were partially alleviated by metformin. Taken together, our findings support the notion that AMPK activation upregulates trophoblast glucose uptake by stimulating GLUT3 translocation, which is beneficial for viability. Thus, the modulation of glucose metabolism in trophoblasts by targeting AMPK might ameliorate the adverse intrauterine environment caused by GDM.

## Introduction

Gestational diabetes mellitus (GDM), which is defined as glucose intolerance during pregnancy, affects up to 20% of all pregnancies (American Diabetes Association, 2018) and is associated with maternal obesity and the subsequent development of type 2 diabetes (T2D) (Chu et al., 2007). Moreover, the fetuses of mothers with GDM are often large for gestational age (LGA) *in utero* and are also predisposed to T2D and metabolic syndrome later in life (Boerschmann et al., 2010; Deierlein et al., 2011; Tam et al., 2017). The mechanisms that lead to the increased risk of subsequent metabolic disorders in both pregnant women with GDM and their offspring are not fully understood, but abnormal placental metabolism of glucose has increasingly been implicated in this process.

The fetal programming for diseases in adulthood is strongly associated with the *in utero* environment, which is tightly related to the maternal-to-fetal transfer of nutrients and other substances (Dabelea and Crume, 2011). An example is the observation of the alterations in the ovine fetal cardiac morphometry in response to maternal nutrition restriction or maternal overfeeding affecting fetal organ development (Huang et al., 2010; Fan et al., 2011; Ge et al., 2013; Kandadi et al., 2013). The human hemochorial placenta, which is an organ that develops from blastocysts, is unique and provides an interface for the exchange of nutrients and metabolites between the mother and fetus (Jansson and Powell, 2007). The placental transfer of glucose is mediated by facilitated diffusion, and the net transport of glucose is therefore highly dependent on the blood glucose gradient across the placenta. Placental trophoblasts undergo substantial and relatively constant aerobic glycolysis (Bax and Bloxam, 1997), which indicates that glucose is the primary energy substrate used by trophoblasts. Notably, the placenta, which is the largest fetal organ at birth (Coppoletta and Wolbach, 1933), has a high blood supply (45 mL/kg blood) (Kakkilaya et al., 2008). Therefore, the placenta has long been hypothesized to play a role in moderating fluctuations in the blood glucose levels in the maternal and fetal circulation through glucose uptake and placental utilization (Illsley, 2000). Compromised glucose uptake by trophoblasts might contribute to an excessive net influx of glucose into the fetal circulation and could thus lead to metabolic disturbances that affect fetal growth.

Glucose uptake and transport by placental trophoblasts is mediated by key facilitative glucose transporters (GLUTs), primarily GLUT1 and GLUT3 (Zhou and Bondy, 1993). GLUT1 is highly expressed in mammalian placental syncytial trophoblasts in the human placenta, whereas GLUT3 exhibits both a higher affinity for glucose and a greater transport capacity than GLUT1 (Simpson et al., 2008). GLUT1 is a ubiquitous isoform expressed in almost all tissues examined thus far and is regarded as the constitutive form of the transporter (Illsley, 2000). In addition, GLUT1 is the major glucose transporter in the human placenta for basal glucose transport (Leonce et al., 2006). In addition, aberrant expression of GLUT1 in the placentas of women with GDM has been demonstrated in some, but not all, studies (Gaither et al., 1999; Jansson et al., 2001). In contrast, GLUT3 is highly expressed in tissues and organs with specific glucose requirements (Vannucci et al., 1997; Deng D. et al., 2015), such as neurons and glia. In addition, GLUT3, as an integral membrane protein, is involved into the ER and ER membranes after the post-transcriptional translation (Brown et al., 2011). In other words, GLUT3 exists by binding membrane structure all the time. Animal experiments have demonstrated that the expression of placental GLUT3 responds to streptozotocin (STZ)-induced maternal hyperglycemia (Boileau et al., 1995), which indicates that aberrant expression patterns and/or dysfunction of GLUT3 in the placenta might be involved in disturbances in glucose metabolism and thus in the development of GDM. AMP-activated protein kinase (AMPK) stimulates glucose uptake in various tissues (Kurth-Kraczek et al., 1999; Yamauchi et al., 2002). The activation of AMPK in rodents with insulin resistance or T2D increases skeletal muscle glucose uptake via an insulin-independent mechanism (Hayashi et al., 1998; Bergeron et al., 2001; Song et al., 2002). Therefore, the AMPK agonist metformin (MET) is widely used as a first-line treatment for T2D (Musi et al., 2002). A recent study demonstrated that AMPK is downregulated in the placentas of women with GDM (Yao et al., 2017), which implies that the uptake of glucose by trophoblasts might be abnormal. In addition to the well-characterized mechanism underlying the AMPK-mediated regulation of GLUT4-mediated glucose uptake in skeletal muscle cells and cardiomyocytes (Kurth-Kraczek et al., 1999; Li et al., 2004), the AMPK-mediated regulation of GLUT3 trafficking has also been observed in neurons (Iwabuchi and Kawahara, 2013); however, few studies have investigated the regulation of GLUT3 in the human placenta or trophoblasts.

The overall aims of this study were to explore the regulatory role of AMPK in the subcellular redistribution of GLUT3 and the consequent uptake of glucose by placental trophoblasts and to determine whether this function is important for buffering the excessive blood glucose gradient between the maternal and fetal circulation.

## Materials and Methods

### Patient Recruitment and Tissue Collection

Thirty-two GDM-complicated and 30 healthy full-term singleton pregnancies were included in this study. The diagnosis of GDM was established based on the results from a 75-g oral glucose tolerance test (OGTT) conducted between gestational weeks 24 and 28 in accordance with the International Association of the Diabetes and Pregnancy Study Groups (IADPSG) criteria. Patients with pre-existing diabetes or any apparent major disease or who conceived through *in vitro* fertilization were excluded. The protocol was approved by the Ethics Committee of The First Affiliated Hospital of Chongqing Medical University (2016–40–2), and written informed consent was obtained from all the subjects. All procedures were performed in accordance with the principles stated in the Declaration of Helsinki. The samples were collected as described previously (Luo et al., 2011).

### Cell Culture

HTR8/SVneo cells were kindly donated by Dr. Charles Graham (Queen’s University, Canada), and Jar and Jeg-3 cells were obtained from the Cell Bank of the Chinese Academy of Sciences (Shanghai, China). HTR8/SVneo and Jar cells were cultured in Roswell Park Memorial Institute (RPMI) 1640 medium (Thermo Fisher Scientific, United States) supplemented with 10% fetal bovine serum (FBS) (Gibco, United States), whereas Jeg-3 cells were cultured in DMEM/F12 medium (Thermo Fisher Scientific) supplemented with 10% FBS. BeWo cells (ATCC, United States) were cultured in 10% FBS-enriched Ham’s F-12K medium (Gibco) supplemented with penicillin (100 units/mL) and streptomycin (100 μg/mL). The cells were maintained at 37°C under humidified conditions with 5% CO_2_ in air. AICAR and forskolin were purchased from Selleck (Houston, TX, United States), and compound C was purchased from Sigma. HTR8/SVneo cells were seeded, cultured to 70% confluence and then treated with 11.1 mM D-glucose combined with 18.9 mM mannitol (osmotic control group), 30 mM glucose (high-glucose group), or 11.1 mM D-glucose (control group) for 24 h. Subsequently, 100 μM AICAR was added to the medium, and the cells were incubated for another 24 h. After treatment, a glucose uptake assay was performed (Figure 3). The syncytialization of BeWo cells was induced by incubation with 50 μM forskolin for 24 h (Rothbauer et al., 2017), and the cells were then incubated with fresh medium containing 30 mM glucose (high-glucose group), 7 mM D-glucose + 23 mM mannitol (osmotic control group), or 7 mM D-glucose (control group). After 30 min, 10 μM AICAR or vehicle was added, and the cells were incubated for 24 h, collected with cell lysis buffer and subjected to Western blotting analysis.

### Membrane Protein Isolation

Membrane proteins were isolated using a Minute^TM^ Plasma Membrane Protein Isolation Kit (Invent Biotechnologies, United States) according to the manufacturer’s instructions. The isolated membrane proteins were then dissolved in denaturing protein solubilization reagent (Invent Biotechnologies, United States). By using this kit, we were able to separate the plasma membrane, nucleus, and cytosol, which contains organelles.

### Immunofluorescence

Immunofluorescence staining was performed as previously described. A primary antibody against AMPK (#2532) was purchased from Cell Signaling, and GLUT1 (21829-1-AP) and GLUT3 (20403-1-AP) primary antibodies were purchased from the Proteintech. Primary antibody against cytokeratin 7 (sc-23876) were purchased from Santa Cruz, and a primary antibody against Ki67 (AF0198) was purchased from Affinity. Fluorescein isothiocyanate-conjugated goat anti-rabbit and anti-mouse antibodies (Santa Cruz) were used as secondary antibodies. The nuclei were counterstained with DAPI (1 mg/mL). The PM was counterstained with DiI (Beyotime), which is a lipophilic membrane stain that diffuses laterally to stain the entire cell and is weakly fluorescent until incorporated into membranes. Images were acquired using an Olympus BMX-60 microscope equipped with a Sensicam cooled charge-coupled device camera (Cooke, United States) or an A1_R_^+^/A1^+^ confocal microscopy (Nikon, Japan). The immunofluorescence data were quantified using ImageJ software, and the quantification of the target protein was based on five fields randomly selected from each slide. The intensity and cells in every field was calculated. The average intensity in each cell was calculated and used for analysis.

### Terminal Deoxynucleotidyl Transferase dUTP Nick End Labeling Staining

Frozen slides of human placentas were incubated with an *In Situ* Cell Death Detection Kit-TUNEL (TMR red, Roche, Schweiz) at 37°C for 1 h following the manufacturer’s instructions to detect DNA-damaged cells (Bouitbir et al., 2016; Lin et al., 2017). The slides were then stained with DAPI for 10 min (Vector Laboratories, United States), and images were captured with an EVOS FL Automicroscope (Life Technologies, United States).

### Seahorse Metabolic Assay

An extracellular flux analyzer XFe96 (Agilent Technologies, United States) was utilized to test the effects of AMPK and compound C on cellular metabolism in HTR8/SVneo cells (Miao et al., 2019; Zhang et al., 2019; Niu et al., 2021). The following assay medium was prepared before the test: Seahorse XF Base Medium containing 2 mM glutamine (Gibco, United States), 10 mM glucose (Gibco, United States), and 1 mM pyruvate (Sigma-Aldrich, United States). The pH was adjusted to 7.4 at 37°C, and a 0.22-μm filter was used for sterilization. A total of 1 × 10^4^ WT HTR8/SVneo cells were seeded per well in 96-well plates designed for the instrument and incubated overnight at 37°C. The cells were then treated with 0.1% DMSO, 200 μM AICAR and 20 μM compound C for 24 h. The growth medium was replaced with the assay medium 1 h prior to detection, and the cells maintained at 37°C in an incubator without CO_2_. The oxygen consumption rate (OCR) was measured through sequential injections of 1 μM oligomycin, 0.5 μM FCCP, and 0.5 μM rotenone/antimycin A (Seahorse XF Cell Mito Stress Test Kit, Agilent, Santa Clara, CA, United States).

### Detection of Cell Death on Villous Explants

First-trimester placental villi samples were collected from healthy women undergoing elective surgical termination of their pregnancies after 6–9 weeks of gestation. These samples were maintained in cold sterile saline and dissected into explants (1–2 mm in diameter). Prior to explant implantation, growth factor-reduced Matrigel (Corning, United States) mixed with serum-free DMEM/F12 medium (Life Technologies, United States) at a ratio of 1:9 was added to 48-well culture plates (BIOFIL, China) and incubated at 37°C with 5% CO_2_ for 8 h to obtain a gel. Subsequently, 600 μL of DMEM/F12 medium with 10% FBS, 100 U/mL penicillin, 100 μg/mL streptomycin and 0.1% ethidium homodimer-II (EthDII, 1 mM in DMSO, Thermo Fisher Scientific E3599, United States) was added to the 48-well culture plates. EthDII is a high affinity fluorescent nucleic acid stain that is weakly fluorescent until bound to DNA, and after binding, the stain emits red fluorescence. The explants were cultured at 37°C with 5% CO_2_ for 15 h and then observed and recorded using an EVOS FL Auto microscope (Life Technologies, United States). Only explants showing successful initiation of trophoblast cell outgrowth were selected and incubated with 0.3% DMSO and 200 μM AICAR for 48 h. The villi outgrowing area and number of dead cells were measured after 0 and 48 h of treatment. The relative cell death percentage was calculated by dividing the cell number by the area of villus outgrowth.

### Flow Cytometry

Cell apoptosis was measured by flow cytometry (Recuero et al., 2009; Lin et al., 2014; Zhou et al., 2019) using a protocol that was previously established by our group (Deng Q. et al., 2015). Briefly, cells (>1 × 10^6^) were collected after treatment by centrifugation at 1,000 rpm for 5 min and resuspended in 400 μL of PBS. The cells were then stained with Annexin V-FITC (blank group) or Annexin V-PE-A (shSLC2A3 and shNC groups) and detected using a FACScan flow cytometer (CytoFLEX, China).

### Measurement of Glycolytic Intermediates by LC-MS/MS

#### Metabolite Extraction From Placental Samples

Placental samples in quintuplicate were collected and sent for metabolon-associated energy metabolism analysis (Applied Protein Technology, Shanghai, China). A homogenate of 100 mg of sample mixed with 1 mL of cold methanol/acetonitrile/H2O (2:2:1, v/v/v) was sonicated at a low temperature (30 min/once, twice) and then centrifuged for 20 min (14,000 *g*, 4°C). The supernatant was dried in a vacuum centrifuge. The dried samples were dissolved in 100 μL of acetonitrile/water (1:1, v/v), adequately vortexed and then centrifuged (14,000 rpm, 4°C, 15 min). The supernatants were collected for LC-MS/MS analysis.

#### Metabolite Profiling Analysis

Analyses were performed using a 1290 Infinity ultrahigh-performance liquid chromatograph (Agilent Technologies, United States) coupled to a Sciex 5500 QTRAP system (AB, United States) using an ACQUITY UPLC BEH Amide column (2.1 × 100 mm, 1.7 μm, Waters MS Technologies, Manchester, United Kingdom). The mobile phase consisted of solvent A (15 mM CH_3_COONH_4_ in water) and solvent B (acetonitrile). The column temperature was 45°C, and the flow rate was 400 μL/min. A 4-μL aliquot of each sample was injected. The gradient program was the following: decrease from 90 to 40% from 0 to 18 min, increase to 90% over 0.1 min and maintained at 90% from 18.1 to 23 min. Quality control (QC) samples prepared from the pooled samples were placed into the analysis sequence at regular intervals (one QC sample after every 5 samples). MS/MS analysis (multiple reaction monitoring, MRM) was performed in the electrospray ionization (ESI) negative mode. The conditions were set as follows: source temperature, 450°C; ion source gas 1, 45; ion source gas 2, 45; curtain gas (CUR), 30; and ion spray voltage floating, -4500 V. Ion pairs were detected using the MRM mode. The standards used to allow the assessment of glycolytic intermediates and the details of the standard curves are described in Supplementary Tables 1, 2. All standards were purchased from Sigma-Aldrich (D-glucose 6-phosphate, V900924; beta-D-fructose 6-phosphate, dihydroxyacetone phosphate, D7137; 344342; phosphoenolpyruvate, P0564; pyruvate, 107360; lactate, L1750; ADP, 01905; and ATP, A7699). The sequence order of all the samples was randomized to avoid interference due to system instability.

The target metabolite signals were matched with those of the standards. The detection of an obvious main peak in line with the retention time (RT) of a standard indicated that the substance could be detected in the sample. In the MRM method, the parent ion pair was set, and the parent ion of each substance was fixed. Each compound has a charge (+H) and was fragmented into several product ions. We selected one of the quantitatively stable fragment ion isomers. Qualitative analysis was then performed by liquid chromatography, which could separate these product ions according to their polarities and different RTs. Metabolites were then identified and quantified using MultiQuant software (AB SCIEX, United Kingdom), and the RT was corrected based on the standards. The relative ion intensity was normalized to the sum of the peak area of a sample.

### 2-Deoxy-D-[^3^H] Glucose Uptake Assay

Wild-type (WT), shNC-transfected and shGLUT3-transfected cells were seeded in 12-well plates at a density of 8 × 10^4^ cells/well and cultured to 70% confluence. Prior to experimentation, the cells were serum deprived for 2 h in glucose-free medium and preincubated for 20 min at 37°C in 1 mL of warm Krebs–Ringer bicarbonate (KRH) buffer (25 mM HEPES-NaOH [pH 7.4], 120 mM NaCl, 5 mM KCl, 1.2 mM MgSO_4_, 1.3 mM CaCl_2_, and 1.3 mM KH_2_PO_4_) to acclimatize the cells and deplete cellular glucose. Glucose uptake was initiated by the addition of 0.5 mL of KRH buffer containing 0.25 μCi/mL 2-deoxy-d-[^3^H] glucose (Perkin Elmer, United States) and 50 μM 2-deoxy-d-glucose followed by a 1-h incubation at 37°C, and glucose uptake was stopped by removal of the buffer followed by three washes with ice-cold KRH buffer. The cells were lysed (0.5 M NaOH and 0.1% SDS), and the amount of labeled glucose taken up was determined using a multipurpose scintillation counter (Beckman Coulter, United States). Total protein was determined by the Bradford method using bovine serum albumin (BSA) as the standard. Radioactive glucose uptake was expressed as nmol/μg protein or Bq/mg protein.

### Glucose Uptake Assay

The fluorescent glucose analog 2-(N-[7-nitrobenz-2-oxa-1,3-diazol-4-yl] amino)-2-deoxy-d-glucose (2-NBDG) (Thermo Fisher Scientific, United States) was used to measure the uptake of glucose by HTR8/SVneo cells. After the above-described treatments, the cells were washed twice with PBS and incubated for 30 min in the absence or presence of 100 μM 2-NBDG in KRPH buffer (118 mM NaCl, 5 mM KCl, 1.2 mM KH_2_PO_4_, 1.3 mM CaCl_2_, 1.2 mM MgSO_4_, and 30 mM HEPES [pH 7.4]). The cells were then washed twice with cold PBS, trypsinized, and resuspended in ice-cold PBS. The fluorescence intensity was measured using a flow cytometer (BD Biosciences, United States) at 535/485 nm and corrected based on the autofluorescence of unlabeled cells.

### Real-Time Quantitative Polymerase Chain Reaction

Total RNA was extracted using TRIzol reagent (Invitrogen, United States) according to the manufacturer’s instructions (Deng Q. et al., 2015). The RNA concentrations and purity were measured using a NanoDrop ND1000 spectrophotometer (Thermo Fisher Scientific, Australia). The RNA was converted into cDNA using a Transcriptor First-strand cDNA synthesis kit (Roche, United States). RT-qPCR was performed using a CFX96 Real-Time PCR Detection System (Bio-Rad, Australia) with SYBR Green (Roche, Germany). The sequences of the primers used for RT-qPCR are presented in Supplementary Table 3. The mean threshold cycle (CT) values were normalized to those of β-actin, and the relative mRNA level of AMPK was calculated using the 2^–ΔΔ^ CT method.

### shRNA Transfection

shRNA targeting GLUT3 (shGLUT3; 5′-GGGCATCGTTGTTG GAATTCT-3′) and a scrambled shRNA (shNC) were designed and synthesized by GenePharma (Shanghai, China). 293FT cells were cotransfected with LV3-GFP-Puro packaging vectors using Lipofectamine 2000 (Invitrogen, United States) to generate lentiviral particles. The shRNAs were transfected into HTR8/SVneo trophoblasts using 5 μg/mL polybrene according to the manufacturer’s recommended protocol. The transfected cells were then screened with 5 μg/mL puromycin.

### Genotyping of the Leptin Receptor^db^ Mutation

Leptin Receptor^db^ mice lack the functional, full-length Ob-Rb leptin receptor. Two microliters of HotSHOT DNA was combined with 23 mL of the PCR mixture. A total of 500 nM of each primer (forward: 5′-AGAACGGAC ACTCTTTGAAGTCTC-3′; reverse: 5′-CATTCAAACCATA GTTTAGGTTTGTGT-3′) was combined with PCR buffer, 2 mM MgCl_2_, 0.2 mM dATP, 0.2 mM dCTP, 0.2 mM dGTP, and 0.2 mM dTTP (KAPA2G Robust HS; Kapa Biosystems, Cape Town, South Africa) to obtain a volume of 25 μL. Amplification was performed with a T100^TM^ Thermal Cycler system (Bio-Rad, Singapore). The PCR product obtained with the 25-μL mixture was digested by the direct addition of 25 μL of 1 × digestion cocktail containing 19 μL of water, 5 μL of 10 × CutSmart Buffer (New England Biolabs, Ipswich, MA, United States), and 1 μL of *Rsa*I restriction enzyme (New England Biolabs) and overnight incubation of the mixture at 37°C. The digested products (50 μL) were analyzed in 4% agarose (Takara Biomedicals, Osaka, Japan) with 1 × TAE buffer containing 0.05% (v/v) GoldView^TM^. Digestion with *Rsa*I yielded 135-bp fragments in + / + mice and 135-, 108- and 27-bp fragments in heterozygotic db/ + mice.

### Oral Glucose Tolerance Test

The mice were fasted for 6 h and then subjected to the OGTT using a Roche Glucose Analyzer (Indianapolis, IN, United States). On gestational day (GD) 16.5, conscious unrestrained mice were orally administered glucose (2 g/kg body weight), and their blood glucose levels in tail pricks performed 0, 30, 60, and 120 min after glucose loading were measured.

### Plasma Analysis

Plasma insulin ELISAs were performed according to the manufacturer’s instructions (Mouse Insulin ELISA; Shanghai Jianglai Biotech, Shanghai, China). The insulin resistance score was determined using the homeostatic model assessment of insulin resistance (HOMA-IR) and was calculated as (fasting plasma glucose × fasting immunoreactive insulin)/22.5; insulin sensitivity was determined by the homeostatic model assessment of insulin sensitivity (HOMA-IS) and calculated as 1/HOMA-IR; and β-cell function was assessed by the homeostatic model assessment of β-cell function (HOMA-β) and calculated as [(20 × fasting immunoreactive insulin)/(fasting plasma glucose −3.5)]%.

### Experimental *db/* + Animals

A 8-week-old db/ + mice were obtained from the Model Animal Research Center of Nanjing University (Nanjing, China). The animals were maintained under a 12-h light/12-h dark cycle in a controlled environment with access to water *ad libitum*. All experimental procedures were completed in accordance with the National Institutes of Health (NIH) Guide for the Care and Use of Laboratory Animals. The procedures were approved by the Medical Ethics Committee of The First Affiliated Hospital of Chongqing Medical University (2016–40–2). Genotyping was performed as described previously with some modifications (Truett et al., 2000; Yamashita et al., 2001).

### High-Fat Diet-Induced Mouse Gestational Diabetes Mellitus Model

A 12-week-old C57BL/6J female mice were randomly fed either (i) an AIN-93G control diet (Research Diets, United States) consisting of 4.73 kcal/g (21.0% protein, 74% carbohydrates, and 5% fat) or (ii) a D12451 high-fat diet (Research Diets, United States) consisting of 4.73 kcal/g (20% protein, 35% carbohydrates, and 45% fat). After one week of dietary intervention, females were bred overnight with age-matched C57BL/6J male mice, and the time at which a copulatory plug appeared the following morning was designated GD0.5. The pregnant mice continued to be fed their respective diets throughout pregnancy. The female mice administered the HFD received either 600 mg/kg/day MET or vehicle (HFD group) via oral gavage daily from GD11.5 to GD18.5.

### Western Blotting

Western blotting was performed as previously reported (Wang et al., 2010; Tong et al., 2013). Placental tissue was homogenized in ice-cold lysis buffer in microfuge tubes containing a protease and phosphatase inhibitor cocktail (Sigma-Aldrich PPC1010) for 10 min. The medium in the HTR8/SVneo cell culture dishes was discarded, and the cells were washed three times with ice-cold PBS. The cells were then incubated with ice-cold lysis buffer for 10 min, scraped using a cold plastic cell scraper and collected in microfuge tubes. Both the placental tissue and HTR8/SVneo cells were treated five times with an ultrasonic cell crusher and centrifuged at 14,000 *g* and 4°C for 12 min. The supernatant was collected in a fresh tube and placed on ice. The protein concentration was determined using the Bradford method (Bio-Rad, Hercules, CA, United States). Placentally homogenized proteins (30 μg in each lane) were then separated by sodium dodecyl sulfate polyacrylamide gel electrophoresis (SDS-PAGE) and transferred to polyvinylidene difluoride (PVDF) membranes. Rabbit polyclonal antibodies against AMPKα (#2532), phospho-AMPKα (Thr172) (#2535), acetyl-CoA carboxylase (ACC) (#3676), phospho-ACC (p-ACC^Ser79^) (#11818), Na^+^/K^+^-ATPase (#23565), Bax2 (#2772), Bcl2 (#4223), p53 (#2524) and p-p53^Ser15^ (#9284) and horseradish peroxidase-linked secondary antibodies were purchased from Cell Signaling, and the GLUT3 (sc-74399) antibody was purchased from Santa Cruz. Protein kinase B (Akt) (ab8805) and phospho-protein kinase B (p-Akt^Ser473^) (ab81283) antibodies were purchased from Abcam, and the Ki67 (AF0198) antibody was purchased from Affinity. The Western blots were quantified using ImageJ software. The band intensity was extracted and used for statistical analysis. For PM GLUTs/cytoplasmic GLUTs analysis, the PM-associated GLUT level was normalized to Na^+^-K^+^-ATPase and the cytoplasmic GLUT level was normalized to either GAPDH or β-actin, then the former was divided by the latter to obtain the PM GLUTs/cytoplasmic GLUTs ratio.

### Statistical Analyses

GraphPad Prism 8.0 was used for the statistical analyses. All the clinical demographic data (i.e., age and body mass index (BMI)) of the subjects were found to be normally distributed variables, are summarized as the means ± standard deviations (SDs) and were analyzed using Student’s *t*-test. The metabolic data were analyzed using Student’s *t*-test to identify metabolites with significant differences between the normal and GDM groups. For the other data, normally distributed variables are summarized as the means ± standard errors of the mean (SEMs) and were analyzed with Student’s *t*-test (between two groups) or ordinary one-way ANOVA (among multiple groups); in contrast, non-normally distributed variables are presented as the medians (interquartile ranges, IQRs) and were analyzed using the Mann-Whitney test (between two groups) or Kruskal-Wallis test (among multiple groups). In the statistical graphs included in all the figures, the data that are presented as the means ± SEMs are annotated in normal font, and the data presented as the medians (IQRs) are shown in bold font, unless otherwise stated.

All statistical tests were two-sided, and *P* < *0.05* indicated statistical significance unless otherwise stated.

## Results

### Subject Characteristics

The clinical data of all the participants are summarized in Supplementary Table 4. The pregnant women in the control and GDM groups had similar maternal ages, BMIs, gestational durations, systolic and diastolic blood pressures, and placental weights. However, the women with GDM exhibited significantly higher parity (1.41 ± 0.50 vs. 1.07 ± 0.25; *p* < 0.005), significantly increased neonatal birth weights (3682 ± 501.6 vs. 3432 ± 389.7 g; *p* < 0.05) and higher fasting, 1-h and 2-h plasma glucose levels (5.29 ± 0.64 vs. 4.49 ± 0.20, 10.13 ± 1.65 vs. 7.21 ± 1.16, and 8.53 ± 1.36 vs. 5.07 ± 0.66 mmol/L, respectively; *p* < 0.0001) compared with those in the control group.

### Glucose Metabolism Is Downregulated in the Placentas of Women With Gestational Diabetes Mellitus

**FIGURE 1:**
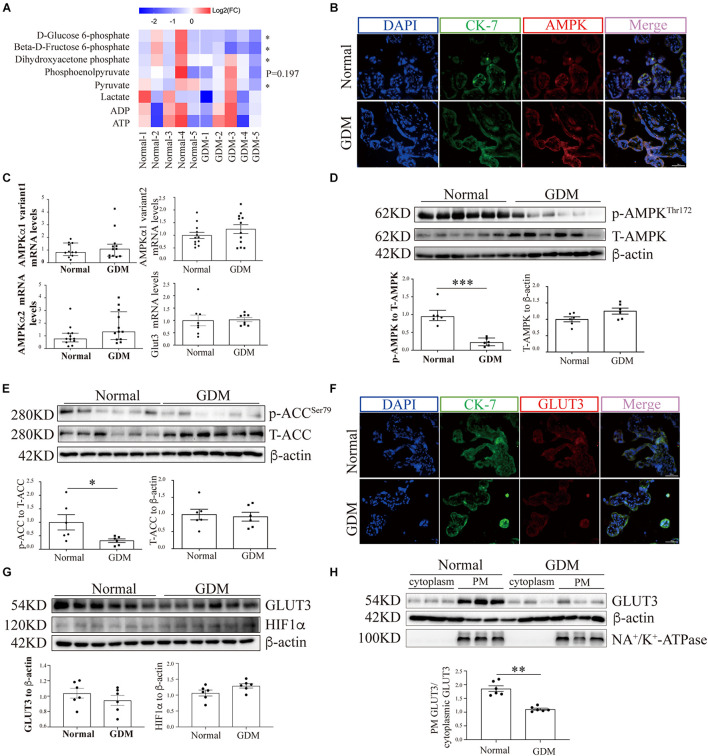
AMP-activated protein kinase signaling and the membrane-bound GLUT3 levels are compromised in GDM-complicated human placentas. **(A)** Heatmaps showing the relative intracellular levels of glycolytic intermediates in normal and GDM-complicated placentas. The fold change (FC) represents the ratio of the ion intensity of each compound in each sample to the average ion intensity of the compound in the normal group, and Log2(FC) values were then calculated and are presented in the heatmap. The color indicates the abundance of representative compounds, and the color key is shown at the top of the figure. Student’s *t*-test was used for the analysis; *n* = 5. **(B)** IF staining of AMPK in normal and GDM-complicated human placentas. Extravillous trophoblast cells were stained with CK-7, and nuclei were counterstained with DAPI. Bar: 50 μm. **(C)** mRNA levels of AMPKα and GLUT3 in GDM-complicated and control human placentas; *n* = 8–12/group. **(D)** p-AMPK and AMPK levels in placental tissue from the GDM and control groups. β-Actin was used as a loading control; *n* = 6. **(E)** p-ACC and ACC levels in GDM-complicated and control placentas. β-Actin was used as a loading control; *n* = 6. **(F)** IF staining of GLUT3 in normal and GDM-complicated human placentas. Extravillous trophoblast cells were stained with cytokeratin 7 (CK-7), and nuclei were counterstained with DAPI. Bar: 50 μm. **(G)** GLUT3 and HIF-1α protein levels in GDM-complicated and normal placentas; *n* = 6. **(H)** Levels of PM-bound GLUT3 protein in GDM-complicated and normal placentas. *n* = 6; PM GLUT3: PM GLUT3 expression/Na^+^-K^+^-ATPase expression; cytoplasmic GLUT3: cytoplasmic GLUT3 expression/actin expression. The data that are presented as the means ± SEMs are annotated in normal font, and Student’s *t*-test was used between two groups. The data presented as the medians (IQRs) are shown in bold font and Mann-Whitney test was used between two groups. ^∗^*p* < 0.05, ^∗∗^*p* < 0.01, and ^∗∗∗^*p* < 0.001.

To evaluate the overall glycolytic status, placental tissues collected from normal and GDM pregnancies (*n* = 5) were examined for key intermediates of glycolysis by LC-MS/MS. The results suggested that D-glucose-6-phosphate and beta-D-fructose 6-phosphate were significantly downregulated in the GDM group (Figure 1A). In line with this finding, the downstream metabolic intermediates of glycolysis, including dihydroxyacetone phosphate, pyruvate and lactic acid, tended to be decreased in the GDM group, which confirmed that placental glucose metabolism is compromised in women with GDM.

### The AMP-Activated Protein Kinase Signaling Pathway Is Compromised in the Placentas of Women With Gestational Diabetes Mellitus

The expression of AMPK in term placentas was confirmed by IF staining (Figure 1B), and the AMPK transcription levels in the placenta, as assessed by RT-qPCR, did not differ between GDM and normal pregnancies (Figure 1C). To further evaluate the placental AMPK activity in the context of GDM, the phosphorylated AMPK protein levels were determined by Western blotting. As shown in Figure 1D, significantly lower AMPK phosphorylation was found in the placentas of pregnant women with GDM than in those of women with normal pregnancies. Consistent with this finding, the phosphorylation levels of acetyl-CoA carboxylase (ACC), a direct downstream effector of AMPK, were also significantly reduced in GDM-complicated placentas (Figure 1E).

### Glucose Transporter 3 Translocation and Cellular Viability Are Impaired in the Placentas of Women With Gestational Diabetes Mellitus

**FIGURE 2:**
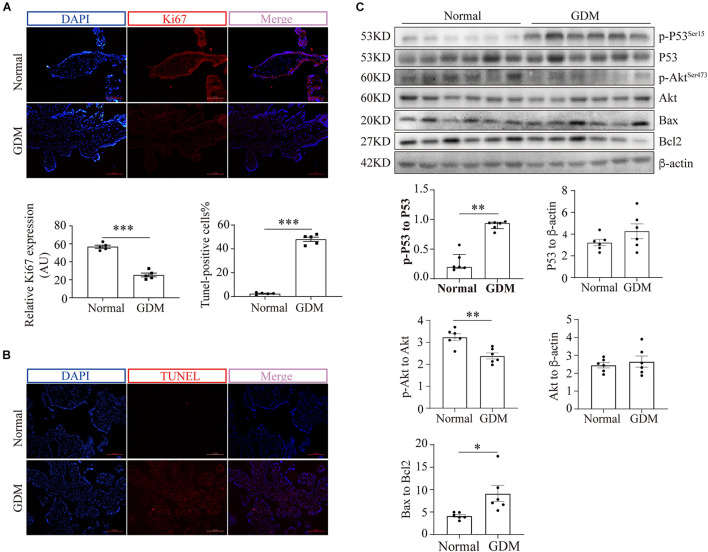
Cell viability is compromised in GDM-complicated human placentas. **(A)** IF staining of Ki67. Bar: 100 μm, *n* = 5. The fluorescence was measured as the intensity per unit area, and the average intensity was calculated for analysis. **(B)** TUNEL staining of normal and GDM-complicated human placentas. Bar: 100 μm, *n* = 5; the percentage of TUNEL-positive cells represents the ratio of the number of TUNEL-positive stained cells to the total cell number. **(C)** p-p53, p53, p-Akt, Akt, Bax and Bcl2 protein levels in GDM-complicated and normal placentas. *n* = 6. The data that are presented as the means ± SEMs are annotated in normal font, and Student’s *t*-test was used between two groups. The data presented as the medians (IQRs) are shown in bold font and Mann-Whitney test was used between two groups. ^∗^*p* < 0.05, ^∗∗^*p* < 0.01, and ^∗∗∗^*p* < 0.001.

To exclude the possibility that GDM-complicated placentas exhibit a more generalized impairment of glucose uptake, we first examined the expression of GLUT1. The results showed that neither the mRNA nor the total protein level of GLUT1 was significantly disturbed in the GDM group; consistently, PM-bound GLUT1 was not altered in the placentas of women with GDM (Supplementary Figure 1). We then assessed the expression of GLUT3 in the placenta. IF staining did not reveal significant differences in placental GLUT3 expression between the GDM and normal groups (Figure 1F). Similarly, no differences in the placental expression of total GLUT3 or hypoxia-inducible factor-1alpha (HIF-1α) were found among the groups (Figure 1G). Intriguingly, the distribution of GLUT3 on the PM was significantly compromised in the placentas of women with GDM (Figure 1H), which indicated that the placental uptake of glucose might be impaired. Therefore, the cell viability in the GDM placentas was evaluated. First, Ki67 expression was examined by IF staining, and the results showed that the expression of Ki67 was significantly downregulated in human GDM placentas compared with normal placentas (Figure 2A). TUNEL staining was then performed, and the number of DNA-damaged cells was markedly increased in GDM placentas (Figure 2B). To validate these findings, proapoptotic and antiapoptotic signaling was further detected by Western blotting. The p-p53-to-p53 ratio, p-Akt level and Bax-to-Bcl2 ratio were significantly elevated in GDM placentas (Figure 2C). These data indicate that the disturbed AMPK-GLUT3 axis in GDM placentas might be correlated with cell viability.

**FIGURE 3:**
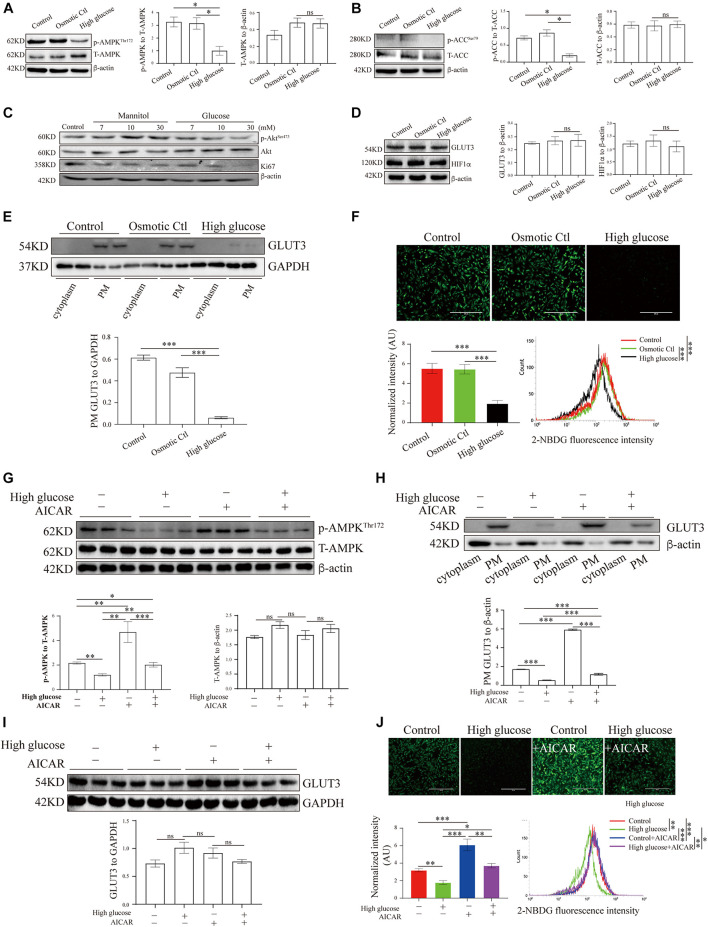
AMP-activated protein kinase signaling and GLUT3 trafficking were manipulated in an *in vitro* trophoblastic GDM model. HTR8/SVneo cells were treated with 11.1 mM D-glucose + 18.9 mM mannitol (osmotic control), 30 mM glucose (high glucose) or 11.1 mM D-glucose (control) and were then subjected to Western blotting analyses of **(A)** the p-AMPK and AMPK levels (*n* = 6), **(B)** the p-ACC and ACC levels (*n* = 6), **(C)** the Ki67, p-Akt and Akt levels (*n* = 6), **(D)** the total GLUT3 and HIF-1α protein levels (*n* = 4) and **(E)** the membranous GLUT3 levels (*n* = 4). PM GLUT3 was normalized to GAPDH. **(F)** Representative fluorescence images from each group after incubation with 2-NBDG for 30 min are shown. Bar: 400 μm. The fluorescence intensity of 2-NBDG taken up by HTR8/SVneo cells was measured by flow cytometry at 488/530 nm (*n* = 4). The intensity of each experiment was calculated using flow cytometry software, and the intensity was normalized to the cell number. **(G)** p-AMPK and AMPK levels in HTR8/SVneo cells treated with vehicle or 100 μM AICAR for 24 h in the presence of a high or normal concentration of glucose (*n* = 6). **(H)** Subcellular GLUT3 distribution after the treatments (*n* = 4). PM GLUT3 was normalized to β-actin. **(I)** Total GLUT3 protein levels (*n* = 6). **(J)** Representative fluorescence images of 2-NBDG obtained from each group after 24 h of treatment with vehicle or AICAR. Bar: 400 μm. The fluorescence intensity of 2-NBDG was measured at 535/485 nm by flow cytometry (*n* = 4). The analysis was in line with that presented in panel **(F)**. The data that are presented as the means ± SEMs are annotated in normal font, and ordinary ANOVA test was used among multiple groups. The data presented as the medians (IQRs) are shown in bold font, and Kruskal-Wallis test was used among multiple groups. ^∗^*p* < 0.05, ^∗∗^*p* < 0.01, and ^∗∗∗^*p* < 0.001.

### Hyperglycemia Inhibits AMP-Activated Protein Kinase Activation, Glucose Transporter 3 Translocation and Glucose Uptake in Trophoblasts *in vitro*

To study the translocation of GLUT3 in trophoblasts, the levels of GLUT3 in BeWo, Jar, Jeg-3 and HTR8/SVneo cells were assessed. Although the subcellular distribution of GLUT1, GLUT3 and GLUT4 did not differ among the cell lines (Supplementary Figure 2A), GLUT1 and GLUT3 were found to be the predominant GLUTs in HTR8/SVneo cells (Supplementary Figure 2B). Moreover, the high-glucose and mannitol treatments notably affected the membranous expression of GLUT3 in syncytialized BeWo cells (Supplementary Figure 2C); this finding indicated that pretreatment with forskolin might induce AMPK activation in BeWo cells (Egawa et al., 2008), which leads to improved PM localization of GLUT3 and thereby to a blunting of the additional high-glucose insult. Therefore, HTR8/SVneo cells were used in the following experiments, and the levels of AMPK signaling and GLUT3 translocation in cells treated with different concentrations of glucose were assessed. The results showed that only 30 mM glucose significantly inhibited AMPK signaling (Supplementary Figures 3A-D), probably because HTR8/SVneo cells are normally cultured under relatively high-glucose conditions. Thus, 30 mM glucose, which has been widely used in studies of diabetes (Bouitbir et al., 2016; Zhong et al., 2019), was selected to stimulate the hyperglycemic response in HTR8/SVneo cells.

These results demonstrate that AMPK phosphorylation was significantly decreased under high-glucose conditions compared with control conditions (Figure 3A), and the phosphorylation of ACC was similarly reduced under high-glucose conditions (Figure 3B). Meanwhile, hyperglycemia significantly suppressed Akt phosphorylation and Ki67 expression in HTR8/SVneo cells (Figure 3C). To further investigate the effects of hyperglycemia on glucose metabolism in trophoblasts, we measured the levels of GLUT3 expression and glucose uptake in hyperglycemic HTR8/SVneo cells. Although neither GLUT3 nor HIF-1α expression was altered under hyperglycemic conditions (Figure 3D), the PM-bound GLUT3 level was significantly reduced by hyperglycemia treatment (Figure 3E and Supplementary Figure 2D), and consistent with this finding, glucose uptake was almost entirely eliminated under high-glucose conditions (Figure 3F).

### AMP-Activated Protein Kinase Regulates Glucose Transporter 3 Translocation, Glucose Uptake and Viability in Human Trophoblasts

To investigate whether the aberrant GLUT3 translocation induced by hyperglycemia is due to AMPK inhibition, the activity of AMPK in HTR8/SVneo cells was modulated with AICAR. AICAR treatment significantly increased the phosphorylation of AMPK (Hall et al., 2018; Figure 3G). In addition, AICAR promoted the increase of PM localization of GLUT3 (Figure 3H), without altering total expression of GLUT3 (Figure 3I). Consistently, the uptake of glucose by HTR8/SVneo cells incubated under hyperglycemic conditions was markedly augmented by AICAR treatment (Figure 3J). These data strongly suggest that the AMPK-induced trafficking of GLUT3 from the cytosol to the PM facilitates the uptake of glucose by trophoblasts.

### AMP-Activated Protein Kinase Regulates the Viability of Trophoblasts Partially Through Energy Metabolism

**FIGURE 4:**
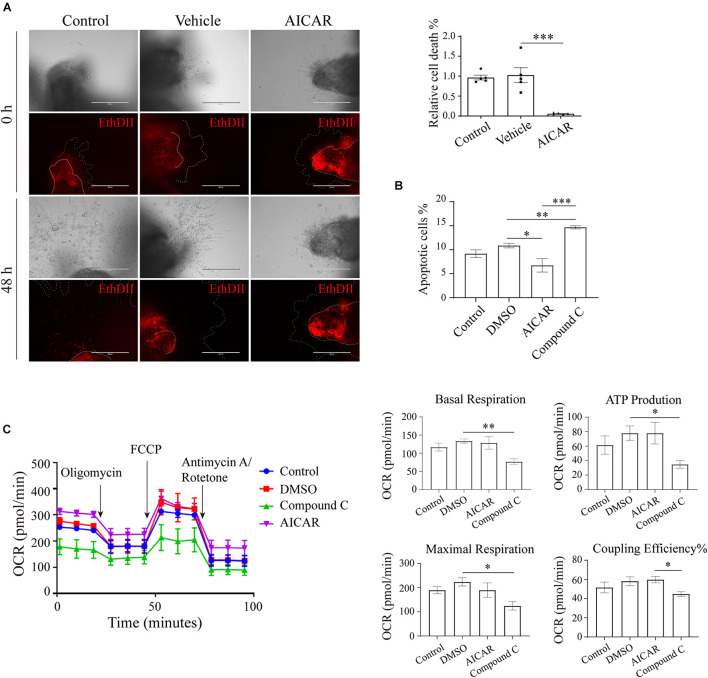
AMP-activated protein kinase regulates viability in an *in vitro* trophoblastic GDM model. **(A)** EthD II-based detection of cell death in villous explants treated with 200 μM AICAR for 48 h. Bar: 400 μM; *n* = 5–27/group. **(B)** The apoptosis of HTR8/SVneo cells after 24 h of incubation with 200 μM AICAR or 10 μM compound C was measured by flow cytometry (*n* = 3); 0.1% DMSO served as the vehicle. **(C)** Oxygen consumption rate (OCR) of HTR8/SVneo cells treated with 200 μM AICAR or 10 μM compound C for 24 h (*n* = 3). The data that are presented as the means ± SEMs are annotated in normal font, and ordinary ANOVA test was used among multiple groups. The data presented as the medians (IQRs) are shown in bold font, and Kruskal-Wallis test was used among multiple groups. ^∗^*p* < 0.05, ^∗∗^*p* < 0.01, and ^∗∗∗^*p* < 0.001.

We further explored the effects of AMPK on trophoblast viability. First, human first-trimester villi collected from normal pregnancies were treated with AICAR, and the death of the cells was monitored. After 48 h of culture, the villi in the control and vehicle groups showed many dead cells in the outgrowth area. However, in the presence of AICAR, cell death was barely detected in the outgrowth area (Figure 4A). Flow cytometry confirmed that AICAR treatment suppressed HTR8/SVneo cell apoptosis, whereas compound C, an AMPK inhibitor, notably elevated cell apoptosis (Figure 4B). These results indicate that AMPK activity is involved in the regulation of trophoblastic viability.

To ascertain whether the impact of the AMPK-GLUT3 axis on trophoblast viability is correlated with energy metabolism, the cellular metabolism of HTR8/SVneo cells was measured using a Seahorse XF Analyzer. As shown in Figure 4C, compound C treatment resulted in a lower oxygen consumption rate (OCR) than that found with the DMSO group, the levels of basal respiration, ATP production and maximal respiration were significantly decreased in the compound C group compared with the DMSO group, and the compound C group also exhibited a lower coupling efficiency than the AICAR group. However, AICAR treatment did not significantly alter cellular metabolism. These facts imply that activation of AMPK with AICAR is not sufficient to boost metabolism because other molecules are not coordinately available to respond to elevated, active AMPK. Whereas inhibition of AMPK with C compound suppresses downstream metabolic functions.

### Intrauterine Hyperglycemia During Pregnancy Impairs AMP-Activated Protein Kinase Activity and Glucose Transporter 3 Translocation in the Mouse Placenta

**FIGURE 5:**
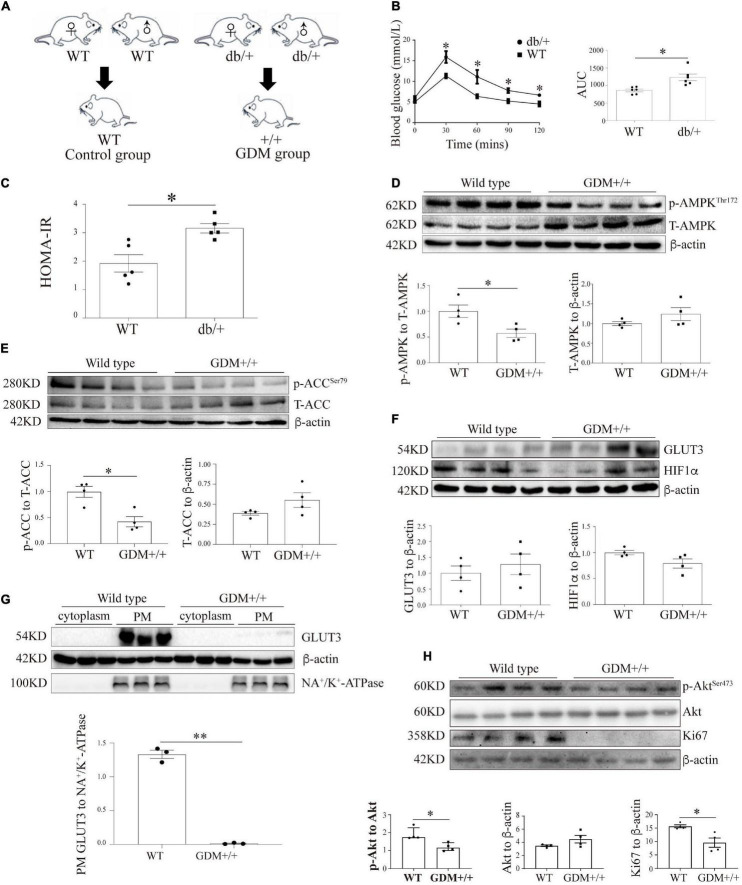
The genetic GDM mouse model is associated with compromised AMPK signaling and altered GLUT3 membrane localization in placental tissue. **(A)** Animal mating plan and study groups. **(B)** OGTT and AUC values (*n* = 6). **(C)** HOMA-IR scores (*n* = 5). **(D)** Placental p-AMPK and AMPK levels in the GDM + / + and WT groups (*n* = 7 from 4 dams). **(E)** Placental p-ACC and ACC levels in the GDM + / + and WT groups (*n* = 7 from 4 dams). **(F)** GLUT3 and HIF-1α protein expression in the GDM and WT placentas (*n* = 7 from 3 dams). **(G)** PM-bound GLUT3 protein levels in GDM + / + and WT mouse placentas (*n* = 7 from 3 dams). PM GLUT3 was normalized to NA^+^/K^+^-ATPase. **(H)** p-Akt, Akt and Ki67 protein levels in GDM + / + and WT mouse placentas (*n* = 4). The data that are presented as the means ± SEMs are annotated in normal font, and Student’s *t*-test was used between two groups while ordinary ANOVA test was used among multiple groups. The data presented as the medians (IQRs) are shown in bold font, and Mann-Whitney test was used between two groups while Kruskal-Wallis test was used among multiple groups. ^∗^*p* < 0.05 and ^∗∗^*p* < 0.001.

LepR^db^ mice lack the functional, full-length Ob-Rb leptin receptor and can exhibit the characteristics of human type 2 diabetes. The breeding of db/ + mice generated + / + mice and siblings with the expected genotypes at a Mendelian ratio (Figure 5A). We subsequently investigated glucose homeostasis in LepR db/ + (db/ +) mice. On GD16.5, the OGTT results and the areas under the curve (AUCs) obtained from the OGTT data demonstrated that the db/ + mice exhibited pronounced glucose intolerance compared with the WT mice (Figure 5B), and a significantly higher HOMA-IR score on GD18.5 was also obtained for the db/ + mice compared with the WT mice (Figure 5C).

To assess the effect of intrauterine hyperglycemia on placental AMPK, the placentas of the WT offspring of the hyperglycemic db/ + dams, which are denoted GDM + / + henceforth, were identified and compared with the placentas of the WT dams. The AMPK phosphorylation level in the GDM + / + placentas was significantly lower than that in the WT control placentas, whereas the total AMPK levels were similar in the two groups (Figure 5D). The phosphorylation level of ACC was markedly reduced in the GDM + / + placentas compared with the WT control placentas (Figure 5E). Moreover, although the placental levels of GLUT3 and HIF-1α did not differ between the GDM + / + and WT control groups (Figure 5F), the PM-bound GLUT3 levels were substantially decreased in the GDM + / + group (Figure 5G). Furthermore, in line with the findings from the human placentas and trophoblast cell lines, Akt signaling and Ki67 expression were significantly compromised in the GDM + / + placentas (Figure 5H).

### Establishment of the HFD-Induced Gestational Diabetes Mellitus Mouse Model

**FIGURE 6:**
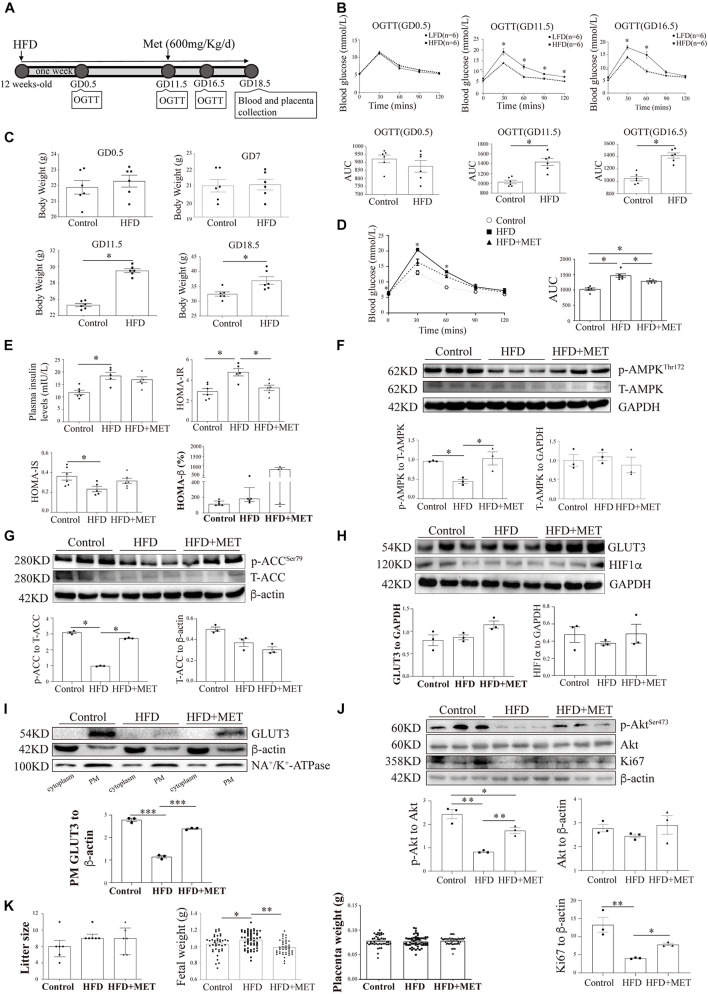
The pharmacological activation of AMPK rescues the shuttling of GLUT3 in the placentas of HFD-induced GDM mice. **(A)** Timeline of the treatments and measurements. **(B)** OGTT and AUC values on GD0.5, GD11.5 and GD16.5 (*n* = 6). **(C)** Body weights on GD0.5, GD7, GD11.5 and GD18.5 (*n* = 6). **(D)** OGTT and AUC values on GD16.5 (*n* = 5–6). **(E)** Plasma insulin levels and HOMA-IR, HOMA-IS and HOMA-β scores on GD18.5 (*n* = 5–6/group). **(F)** Western blot analysis of the p-AMPK and AMPK levels in the placentas from each group (*n* = 8 from 3 dams). **(G)** p-ACC and ACC levels in the placentas (*n* = 8 from 3 dams). **(H)** GLUT3 and HIF-1α protein levels in the mouse placentas (*n* = 8 from 3 dams). **(I)** Membrane-bound GLUT3 protein levels in each group (*n* = 8 from 3 dams). PM GLUT3 was normalized to NA^+^/K^+^-ATPase. **(J)** p-Akt, Akt and Ki67 protein levels in the mouse placentas. **(K)** Litter sizes, fetal birth weights, and placental weights obtained for each group (*n* = 6–68/group). The data that are presented as the means ± SEMs are annotated in normal font, and Student’s *t*-test was used between two groups while ordinary ANOVA test was used among multiple groups. The data presented as the medians (IQRs) are shown in bold font, and Mann-Whitney test was used between two groups while Kruskal-Wallis test was used among multiple groups. *^∗^p* < 0.05, *^∗∗^p* < 0.01, and *^∗∗∗^p* < 0.001.

To further confirm our *in vitro* findings, another GDM murine model was established by feeding female mice an HFD one week before mating and throughout their pregnancy (Figure 6A). On GD0.5, the plasma glucose levels after fasting and after the oral administration of glucose did not differ between the HFD-fed dams and the normal chow-fed controls (Figure 6B). However, on GD11.5 and GD16.5, the basal blood glucose levels in the HFD group increased to 6.85 ± 0.32 mmol/L and 6.42 ± 0.42 mmol/L, respectively, and these values were significantly higher than those found in the control group (5.88 ± 0.17 and 6.10 ± 0.26 mmol/L, respectively); in addition, the areas under the curve based on the OGTT data obtained from the HFD group were significantly higher than those found for the control group (1432 ± 72.52 vs. 1026 ± 35.85 and 1416 ± 42.41 vs. 1043 ± 35.28, respectively). Moreover, on GD0.5 and GD7, the body weight of the control group was similar to that of the HFD group; however, on GD11.5 and GD18.5, the body weight of the HFD group was significantly greater than that of the control group (29.52 ± 0.25 g vs. 25.32 ± 0.20 g and 36.98 ± 1.32 g vs. 32.44 ± 0.73 g, respectively) (Figure 6C). The plasma insulin levels were also significantly higher in the HFD group than in the control group (18.39 ± 0.92 mIU/L vs. 12.36 ± 1.71 mIU/L) (Figure 6E). In accordance, on GD18.5, the HFD group presented a significantly higher HOMA-IR score (4.95 ± 0.25 vs. 3.11 ± 0.51) and a significantly lower HOMA-IS score (0.20 ± 0.01 vs. 0.37 ± 0.06) than the control group. The HOMA-β measurements demonstrated that the function of pancreatic β-cells was comparable between the two groups. These results confirmed that this mouse model exhibited hyperglycemia, increased insulin resistance and decreased insulin sensitivity. All of these alterations in glucose metabolism emerged at the midgestational timepoint, which mimics the timing and phenotype of human GDM.

### Metformin Activates AMP-Activated Protein Kinase and Accelerates Glucose Transporter 3 Translocation in the Placenta of Mice With HFD-Induced Gestational Diabetes Mellitus

To investigate the *in vivo* effects of AMPK activation on placental GLUT3 translocation, 600 mg/kg MET (Yang et al., 2013) was administered through oral gavage daily from GD11.5 to GD18.5. After MET treatment, the plasma glucose level in the HFD group was 5.22 ± 0.10 mmol/L, which was significantly lower than that in the untreated group (6.13 ± 0.37 mmol/L) and comparable to those in the normal chow-fed control group (5.03 ± 0.17 mmol/L) and the group treated with MET alone (5.16 ± 0.16 mmol/L) (Figure 6D). Similarly, on GD18.5, the significant elevation in the HOMA-IR score observed in the HFD group (4.50 ± 0.49 vs. 3.11 ± 0.51 in the control group) was largely alleviated by MET treatment (3.27 ± 0.25), and this effect was not accompanied by notable alterations in insulin secretion, the HOMA-IS score, or the HOMA-β score (Figure 6E). In addition, the administration of MET to HFD-fed mice markedly augmented the placental AMPK phosphorylation level without altering the total AMPK level (Figure 6F), and this finding was further confirmed by examination of the phosphorylation levels of ACC (Figure 6G). Most importantly, although MET did not alter the total GLUT3 or HIF-1α protein levels in the placenta (Figure 6H), it significantly rescued the localization of GLUT3 on the PM, which was altered in the HFD-fed mice (Figure 6I). Moreover, the significant downregulation of Akt and Ki67 in the HFD-fed group was averted by MET treatment (Figure 6J). Additionally, MET treatment rescued the HFD-induced elevation in fetal birth weight without impacting the litter size or placental weight (Figure 6K), which implies that this treatment exerts favorable effects on intrauterine fetal growth.

## Discussion

Despite significant efforts to study the transplacental flux of glucose (Gaither et al., 1999), the correlation between GDM and glucose uptake/metabolism in placental trophoblasts remains to be elucidated. In this study, we observed reduced glucose metabolism in the placentas from GDM-complicated pregnancies, which suggested that the utilization of glucose by the placenta was compromised and that the transport of glucose into the fetal circulation might be consequently increased. Although the impact of GDM on various GLUTs has been investigated (Stanirowski et al., 2017), the involvement of GLUT3 in GDM is underestimated because GLUT3 transports extracellular glucose at an approximately sevenfold faster rate than that observed with GLUT1 (Simpson et al., 2008). We revealed that a 50% reduction in GLUT3 results in a 30% decrease in trophoblast glucose uptake, which is comparable to the effects of the pharmacological inhibition of GLUT1 and GLUT4 by fasentin (Ocaña et al., 2020) (Supplementary Figure 4). These data suggest that GLUT3 might play a major role in transporting glucose into trophoblasts. The present study provides the first evidence demonstrating that GDM is associated with compromised GLUT3 translocation from the cytosol to the PM in trophoblasts. The expression and localization of GLUT3 in trophoblasts are associated with hypoxia (Lee et al., 2015). Therefore, we also examined the HIF-1α levels, and our results suggested that the altered translocation of GLUT3 observed in GDM-complicated placentas and in trophoblasts subjected to high glucose treatment was not due to changes in oxygen tension but to hyperglycemic conditions. High glucose impairs cell viability by suppressing Akt and Ki67 in endothelial progenitor cells (Tian et al., 2020). Here, we found that cell proliferation was impaired in both human and mouse placentas complicated with GDM, whereas apoptosis was enhanced. Consistently, the activation of AMPK in HTR8/SVneo cells ameliorated cell death, whereas the inhibition of AMPK yielded the opposite effects. This evidence suggested that hyperglycemia might impair the viability of trophoblasts by reducing AMPK-GLUT3-dependent glucose metabolism. Moreover, a seahorse assay demonstrated that although the activation of AMPK did not improve energy metabolism, the inhibition of AMPK resulted in significantly lower cellular ATP levels and compromised oxidative phosphorylation, which indicates that basal AMPK activity is needed for energy production and that the beneficial pathway of AMPK on trophoblast survival is independent of ATP production.

AMP-activated protein kinase plays a key role in intracellular energy homeostasis (Garcia and Shaw, 2017), and glucose is the major energy substrate used by trophoblasts (Ericsson et al., 2005). Although GLUT3 expression is regulated by AMPK in astrocytes (Iwabuchi and Kawahara, 2013), the correlations between AMPK and GLUT3 in other cell types remain to be reported. In this study, we found that the AMPK signaling pathway was significantly compromised in placentas from GDM pregnancies, in high glucose-treated trophoblasts, and in placentas from animal models of GDM; these findings are consistent with those recently reported by Yao et al. (2017). Because our data did not show significant differences in either the ADP or ATP levels between the normal and GDM groups, the dephosphorylation of AMPK in GDM-complicated placentas is unlikely to be caused by allosteric regulation (Hardie et al., 1999). Therefore, the upstream kinases that phosphorylate the activation site of AMPK at Thr172, including liver kinase B 1 (LKB1) (Sakamoto et al., 2006), calcium–calmodulin-dependent kinase kinase-β (CaMKKβ) (Woods et al., 2005) and transforming growth factor-β-activated protein kinase-1 (TAK1), should be further investigated to determine the mechanism through which AMPK signaling is inhibited in placentas from GDM pregnancies.

The AMPK-mediated regulation of the translocation of GLUT4 in skeletal muscle cells has been well characterized (Kurth-Kraczek et al., 1999); here, we established an *in vitro* hyperglycemic trophoblast cell model and demonstrated that the trafficking of GLUT3 from the cytosol to the PM is regulated by the activity of AMPK. Although the interaction between AMPK and GLUTs might occur through a tether protein within a protein complex (Bogan et al., 2003), the potential physical interaction between AMPK and GLUT3 proteins needs further validation.

Emerging evidence suggests that the AMPK activator MET might be a better glucose-lowering therapy for GDM than glyburide (Nachum et al., 2017), but whether the pharmacological activation of AMPK regulates placental GLUTs is unclear. This study provides evidence showing that MET treatment increases the membranous expression of GLUT3 in the placentas of murine models of GDM. First, a heterozygous LepR db/ + (db/ +) mouse model with compromised leptin receptor activity was established; this model has long been used as a genetic animal model of GDM (Chick et al., 1970; Kaufmann et al., 1981). Intriguingly, female db/ + mice maintain normal glycemic control in their virgin state but spontaneously develop hyperglycemia after the onset of pregnancy (Ishizuka et al., 1999). Second, an HFD-induced GDM model was developed: in this model, hyperglycemia and insulin resistance are induced late in pregnancy, which mimics the timing of human GDM. Although we have shown that the pharmacological activation of AMPK by MET lowers the blood glucose level and improves intrauterine fetal growth in murine models of GDM, the contribution of trophoblastic GLUT3 to the amelioration of hyperglycemia remains unclear because the administration of MET might also lower the circulatory glucose levels by simultaneously enhancing the GLUT4-mediated uptake of glucose in skeletal muscle cells. Therefore, future studies could utilize genetic models with placenta-specific deficiency in AMPK or GLUT3 to enhance our understanding of the role of the AMPK-mediated translocation of trophoblastic GLUT3 during GDM development. Alternatively, the placenta-specific delivery of AMPK agonists by aptamer-guided nanoparticles could be used to confirm the role of trophoblastic GLUT3 in maintaining glucose homeostasis across the maternal-fetal interface (Zhang et al., 2018).

## Conclusion

**FIGURE 7:**
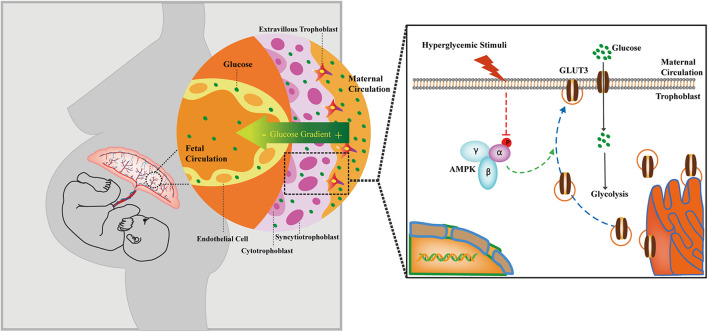
Cartoon representation showing the proposed working model of the trophoblastic AMPK-GLUT3 axis in response to maternal hyperglycemia.

The AMPK signaling pathway is compromised in GDM-complicated pregnancies, and this effect reduces both the trafficking of GLUT3 from the cytoplasm to the PM and the uptake of glucose by trophoblasts (Figure 7). The activation of AMPK stimulates the membranous expression of GLUT3 *in vivo*, and this stimulation promotes the uptake and metabolism of glucose by placental trophoblasts, which results in improving cell viability, lowering the blood glucose level in the maternal circulation and preventing excessive transplacental glucose transport into the fetus, and thus provides a favorable *in utero* environment for fetal growth.

## Data Availability Statement

The raw data supporting the conclusions of this article will be made available by the authors, without undue reservation.

## Ethics Statement

The studies involving human participants were reviewed and approved by the Ethics Committee of The First Affiliated Hospital of Chongqing Medical University. The patients/participants provided their written informed consent to participate in this study. The animal study was reviewed and approved by the Ethics Committee of The First Affiliated Hospital of Chongqing Medical University.

## Author Contributions

CT, HQ, and PB conceived the study. LZ, YW, XYu, FuZ, HF, YZ, PX, XYa, MH, JQ, HW, and XL performed the experiments and analyzed the data. CT, LZ, XYu, and YW wrote the manuscript. LZ, YW, CP, and RG prepared the figures. FY, RS, MK, and PB reviewed and edited the manuscript. CT, JQ, and HQ provided funding for the work. All authors contributed to the article and approved the submitted version.

## Conflict of Interest

The authors declare that the research was conducted in the absence of any commercial or financial relationships that could be construed as a potential conflict of interest.

## Publisher’s Note

All claims expressed in this article are solely those of the authors and do not necessarily represent those of their affiliated organizations, or those of the publisher, the editors and the reviewers. Any product that may be evaluated in this article, or claim that may be made by its manufacturer, is not guaranteed or endorsed by the publisher.

## References

[B1] American Diabetes Association (2018). 2. Classification and diagnosis of diabetes: standards of medical care in diabetes-2018. *Diabetes Care* 41(Suppl. 1), S13–S27. 10.2337/dc18-S002 29222373

[B2] Bax B. E. Bloxam D. L. (1997). Energy metabolism and glycolysis in human placental trophoblast cells during differentiation. *Biochim. Biophys. Acta* 1319 283–292. 9131049 10.1016/s0005-2728(96)00169-7

[B3] Bergeron R. Previs S. F. Cline G. W. Perret P. Russell R. R. III Young L. H. (2001). Effect of 5-aminoimidazole-4-carboxamide-1-beta-D-ribofuranoside infusion on in vivo glucose and lipid metabolism in lean and obese Zucker rats. *Diabetes* 50 1076–1082. 11334411 10.2337/diabetes.50.5.1076

[B4] Boerschmann H. Pfluger M. Henneberger L. Ziegler A. G. Hummel S. (2010). Prevalence and predictors of overweight and insulin resistance in offspring of mothers with gestational diabetes mellitus. *Diabetes Care* 33 1845–1849. 10.2337/dc10-0139 20435793 PMC2909075

[B5] Bogan J. S. Hendon N. McKee A. E. Tsao T. S. Lodish H. F. (2003). Functional cloning of TUG as a regulator of GLUT4 glucose transporter trafficking. *Nature* 425 727–733. 10.1038/nature01989 14562105

[B6] Boileau P. Mrejen C. Girard J. Hauguel-de Mouzon S. (1995). Overexpression of GLUT3 placental glucose transporter in diabetic rats. *J. Clin. Invest.* 96 309–317. 10.1172/JCI118036 7615800 PMC185202

[B7] Bouitbir J. Singh F. Charles A.-L. Schlagowski A.-I. Bonifacio A. Echaniz-Laguna A. (2016). Statins trigger mitochondrial reactive oxygen species-induced apoptosis in glycolytic skeletal muscle. *Antioxid. Redox Signal.* 24 84–98. 10.1089/ars.2014.6190 26414931

[B8] Brown K. Heller D. S. Zamudio S. Illsley N. P. (2011). Glucose transporter 3 (GLUT3) protein expression in human placenta across gestation. *Placenta* 32 1041–1049. 10.1016/j.placenta.2011.09.014 22000473 PMC3272879

[B9] Chick W. L. Lavine R. L. Like A. A. (1970). Studies in the diabetic mutant mouse. V. Glucose tolerance in mice homozygous and heterozygous for the diabetes (db) gene. *Diabetologia* 6 257–262. 5432354 10.1007/BF01212235

[B10] Chu S. Y. Callaghan W. M. Kim S. Y. Schmid C. H. Lau J. England L. J. (2007). Maternal obesity and risk of gestational diabetes mellitus. *Diabetes Care* 30 2070–2076. 10.2337/dc06-2559a 17416786

[B11] Coppoletta J. M. Wolbach S. B. (1933). Body length and organ weights of infants and children: a study of the body length and normal weights of the more important vital organs of the body between birth and twelve years of age. *Am. J. Pathol.* 9 55–70. 19970058 PMC2062747

[B12] Dabelea D. Crume T. (2011). Maternal environment and the transgenerational cycle of obesity and diabetes. *Diabetes* 60 1849–1855. 10.2337/db11-0400 21709280 PMC3121421

[B13] Deierlein A. L. Siega-Riz A. M. Chantala K. Herring A. H. (2011). The association between maternal glucose concentration and child BMI at age 3 years. *Diabetes Care* 34 480–484. 10.2337/dc10-1766 21216858 PMC3024372

[B14] Deng D. Sun P. Yan C. Ke M. Jiang X. Xiong L. (2015). Molecular basis of ligand recognition and transport by glucose transporters. *Nature* 526 391–396. 10.1038/nature14655 26176916

[B15] Deng Q. Chen Y. Yin N. Shan N. Luo X. Tong C. (2015). N-acetylglucosaminyltransferase V inhibits the invasion of trophoblast cells by attenuating MMP2/9 activity in early human pregnancy. *Placenta* 36 1291–1299. 10.1016/j.placenta.2015.08.014 26349781

[B16] Egawa M. Kamata H. Kushiyama A. Sakoda H. Fujishiro M. Horike N. (2008). Long-term forskolin stimulation induces AMPK activation and thereby enhances tight junction formation in human placental trophoblast BeWo cells. *Placenta* 29 1003–1008. 10.1016/j.placenta.2008.09.008 18950855

[B17] Ericsson A. Hamark B. Powell T. L. Jansson T. (2005). Glucose transporter isoform 4 is expressed in the syncytiotrophoblast of first trimester human placenta. *Hum. Reprod.* 20 521–530. 10.1093/humrep/deh596 15528266

[B18] Fan X. Turdi S. Ford S. P. Hua Y. Nijland M. J. Zhu M. (2011). Influence of gestational overfeeding on cardiac morphometry and hypertrophic protein markers in fetal sheep. *J. Nutr. Biochem.* 22 30–37. 10.1016/j.jnutbio.2009.11.006 20188535 PMC2901772

[B19] Gaither K. Quraishi A. N. Illsley N. P. (1999). Diabetes alters the expression and activity of the human placental GLUT1 glucose transporter. *J. Clin. Endocrinol. Metab.* 84 695–701. 10.1210/jcem.84.2.5438 10022440

[B20] Garcia D. Shaw R. J. (2017). AMPK: mechanisms of cellular energy sensing and restoration of metabolic balance. *Mol. Cell* 66 789–800. 10.1016/j.molcel.2017.05.032 28622524 PMC5553560

[B21] Ge W. Hu N. George L. A. Ford S. P. Nathanielsz P. W. Wang X.-M. (2013). Maternal nutrient restriction predisposes ventricular remodeling in adult sheep offspring. *J. Nutr. Biochem.* 24 1258–1265. 10.1016/j.jnutbio.2012.10.001 23333094 PMC3633637

[B22] Hall D. T. Griss T. Ma J. F. Sanchez B. J. Sadek J. Tremblay A. M. K. (2018). The AMPK agonist 5-aminoimidazole-4-carboxamide ribonucleotide (AICAR), but not metformin, prevents inflammation-associated cachectic muscle wasting. *EMBO Mol. Med.* 10:e8307. 10.15252/emmm.201708307 29844217 PMC6034131

[B23] Hardie D. G. Salt I. P. Hawley S. A. Davies S. P. (1999). AMP-activated protein kinase: an ultrasensitive system for monitoring cellular energy charge. *Biochem. J.* 338(Pt. 3), 717–722. 10051444 PMC1220108

[B24] Hayashi T. Hirshman M. F. Kurth E. J. Winder W. W. Goodyear L. J. (1998). Evidence for 5’ AMP-activated protein kinase mediation of the effect of muscle contraction on glucose transport. *Diabetes* 47 1369–1373. 9703344 10.2337/diab.47.8.1369

[B25] Huang Y. Yan X. Zhao J. X. Zhu M. J. McCormick R. J. Ford S. P. (2010). Maternal obesity induces fibrosis in fetal myocardium of sheep. *Am. J. Physiol. Endocrinol. Metab.* 299 E968–E975. 10.1152/ajpendo.00434.2010 20876759 PMC3006252

[B26] Illsley N. P. (2000). Glucose transporters in the human placenta. *Placenta* 21 14–12. 10.1053/plac.1999.0448 10692246

[B27] Ishizuka T. Klepcyk P. Liu S. Panko L. Liu S. Gibbs E. M. (1999). Effects of overexpression of human GLUT4 gene on maternal diabetes and fetal growth in spontaneous gestational diabetic C57BLKS/J Lepr(db/+) mice. *Diabetes* 48 1061–1069. 10331411 10.2337/diabetes.48.5.1061

[B28] Iwabuchi S. Kawahara K. (2013). Extracellular ATP-prinoceptor signaling and AMP-activated protein kinase regulate astrocytic glucose transporter 3 in an in vitro ischemia. *Neurochem. Int.* 63 259–268. 10.1016/j.neuint.2013.07.001 23851016

[B29] Jansson T. Powell T. L. (2007). Role of the placenta in fetal programming: underlying mechanisms and potential interventional approaches. *Clin. Sci.* 113 1–13. 10.1042/CS20060339 17536998

[B30] Jansson T. Ekstrand Y. Wennergren M. Powell T. L. (2001). Placental glucose transport in gestational diabetes mellitus. *Am. J. Obstet. Gynecol.* 184 111–116. 10.1067/mob.2001.108075 11174489

[B31] Kakkilaya V. Pramanik A. K. Ibrahim H. Hussein S. (2008). Effect of placental transfusion on the blood volume and clinical outcome of infants born by cesarean section. *Clin. Perinatol.* 35 561–570,xi. 10.1016/j.clp.2008.07.002 18952022

[B32] Kandadi M. R. Hua Y. Zhu M. Turdi S. Nathanielsz P. W. Ford S. P. (2013). Influence of gestational overfeeding on myocardial proinflammatory mediators in fetal sheep heart. *J. Nutr. Biochem.* 24 1982–1990. 10.1016/j.jnutbio.2013.07.003 24075902 PMC3843763

[B33] Kaufmann R. C. Amankwah K. S. Dunaway G. Maroun L. Arbuthnot J. Roddick J. W. Jr. (1981). An animal model of gestational diabetes. *Am. J. Obstet. Gynecol.* 141 479–482. 7294072 10.1016/s0002-9378(15)33263-4

[B34] Kurth-Kraczek E. J. Hirshman M. F. Goodyear L. J. Winder W. W. (1999). 5’ AMP-activated protein kinase activation causes GLUT4 translocation in skeletal muscle. *Diabetes* 48 1667–1671. 10426389 10.2337/diabetes.48.8.1667

[B35] Lee M. Choi S. Y. Kang B. H. Yoo H. J. Song S. Y. Seong I. O. (2015). Differential microscopic finding and glucose transporter 3 expression in terminal chorionic villi among birth weight-discordant twin placentas. *Histol. Histopathol.* 30 955–962. 10.14670/HH-11-600 25728965

[B36] Leonce J. Brockton N. Robinson S. Venkatesan S. Bannister P. Raman V. (2006). Glucose production in the human placenta. *Placenta* 27(Suppl. A), S103–S108. 10.1016/j.placenta.2006.02.006 16618444

[B37] Li J. Hu X. Selvakumar P. Russell R. R. III Cushman S. W. Holman G. D. (2004). Role of the nitric oxide pathway in AMPK-mediated glucose uptake and GLUT4 translocation in heart muscle. *Am. J. Physiol. Endocrinol. Metab.* 287 E834–E841. 10.1152/ajpendo.00234.2004 15265762

[B38] Lin H.-C. Liu S.-Y. Yen E.-Y. Li T.-K. Lai I. R. (2017). microRNA-183 Mediates protective postconditioning of the liver by repressing Apaf-1. *Antioxid. Redox Signal.* 26 583–597. 10.1089/ars.2016.6679 27580417

[B39] Lin R. Angelin A. Da Settimo F. Martini C. Taliani S. Zhu S. (2014). Genetic analysis of dTSPO, an outer mitochondrial membrane protein, reveals its functions in apoptosis, longevity, and Aβ42-induced neurodegeneration. *Aging Cell* 13 507–518. 10.1111/acel.12200 24977274 PMC4076708

[B40] Luo X. Yao Z. W. Qi H. B. Liu D. D. Chen G. Q. Huang S. (2011). Gadd45alpha as an upstream signaling molecule of p38 MAPK triggers oxidative stress-induced sFlt-1 and sEng upregulation in preeclampsia. *Cell Tissue Res.* 344 551–565. 10.1007/s00441-011-1164-z 21519896

[B41] Miao J. Liu J. Niu J. Zhang Y. Shen W. Luo C. (2019). Wnt/beta-catenin/RAS signaling mediates age-related renal fibrosis and is associated with mitochondrial dysfunction. *Aging Cell* 18:e13004. 10.1111/acel.13004 31318148 PMC6718575

[B42] Musi N. Hirshman M. F. Nygren J. Svanfeldt M. Bavenholm P. Rooyackers O. (2002). Metformin increases AMP-activated protein kinase activity in skeletal muscle of subjects with type 2 diabetes. *Diabetes* 51 2074–2081. 12086935 10.2337/diabetes.51.7.2074

[B43] Nachum Z. Zafran N. Salim R. Hissin N. Hasanein J. Gam Ze Letova Y. (2017). Glyburide versus metformin and their combination for the treatment of gestational diabetes mellitus: a randomized controlled study. *Diabetes Care* 40 332–337. 10.2337/dc16-2307 28077460

[B44] Niu M. Zhao F. Bondelid K. Siedlak S. L. Torres S. Fujioka H. (2021). VPS35 D620N knockin mice recapitulate cardinal features of Parkinson’s disease. *Aging Cell* 20:e13347. 10.1111/acel.13347 33745227 PMC8135078

[B45] Ocaña M. C. Martínez-Poveda B. Marí-Beffa M. Quesada A. R. Medina M. Á (2020). Fasentin diminishes endothelial cell proliferation, differentiation and invasion in a glucose metabolism-independent manner. *Sci. Rep.* 10:6132. 10.1038/s41598-020-63232-z 32273578 PMC7145862

[B46] Recuero M. Vicente M. C. Martínez-García A. Ramos M. C. Carmona-Saez P. Sastre I. (2009). A free radical-generating system induces the cholesterol biosynthesis pathway: a role in Alzheimer’s disease. *Aging Cell* 8 128–139. 10.1111/j.1474-9726.2009.00457.x 19239419

[B47] Rothbauer M. Patel N. Gondola H. Siwetz M. Huppertz B. Ertl P. (2017). A comparative study of five physiological key parameters between four different human trophoblast-derived cell lines. *Sci. Rep.* 7:5892. 10.1038/s41598-017-06364-z 28724925 PMC5517571

[B48] Sakamoto K. Zarrinpashneh E. Budas G. R. Pouleur A. C. Dutta A. Prescott A. R. (2006). Deficiency of LKB1 in heart prevents ischemia-mediated activation of AMPKalpha2 but not AMPKalpha1. *Am. J. Physiol. Endocrinol. Metab.* 290 E780–E788. 10.1152/ajpendo.00443.2005 16332922 PMC2128705

[B49] Simpson I. A. Dwyer D. Malide D. Moley K. H. Travis A. Vannucci S. J. (2008). The facilitative glucose transporter GLUT3: 20 years of distinction. *Am. J. Physiol. Endocrinol. Metab.* 295 E242–E253. 10.1152/ajpendo.90388.2008 18577699 PMC2519757

[B50] Song X. M. Fiedler M. Galuska D. Ryder J. W. Fernstrom M. Chibalin A. V. (2002). 5-Aminoimidazole-4-carboxamide ribonucleoside treatment improves glucose homeostasis in insulin-resistant diabetic (ob/ob) mice. *Diabetologia* 45 56–65. 10.1007/s001250200006 11845224

[B51] Stanirowski P. J. Szukiewicz D. Pyzlak M. Abdalla N. Sawicki W. Cendrowski K. (2017). Impact of pre-gestational and gestational diabetes mellitus on the expression of glucose transporters GLUT-1, GLUT-4 and GLUT-9 in human term placenta. *Endocrine* 55 799–808. 10.1007/s12020-016-1202-4 27981520 PMC5316392

[B52] Tam W. H. Ma R. C. W. Ozaki R. Li A. M. Chan M. H. M. Yuen L. Y. (2017). In utero exposure to maternal hyperglycemia increases childhood cardiometabolic risk in offspring. *Diabetes Care* 40 679–686. 10.2337/dc16-2397 28279981 PMC5399651

[B53] Tian D. Xiang Y. Tang Y. Ge Z. Li Q. Zhang Y. (2020). Circ-ADAM9 targeting PTEN and ATG7 promotes autophagy and apoptosis of diabetic endothelial progenitor cells by sponging mir-20a-5p. *Cell Death Dis.* 11:526. 10.1038/s41419-020-02745-x 32661238 PMC7359341

[B54] Tong C. Morrison A. Mattison S. Qian S. Bryniarski M. Rankin B. (2013). Impaired SIRT1 nucleocytoplasmic shuttling in the senescent heart during ischemic stress. *FASEB J.* 27 4332–4342. 10.1096/fj.12-216473 23024374 PMC3804750

[B55] Truett G. E. Heeger P. Mynatt R. L. Truett A. A. Walker J. A. Warman M. L. (2000). Preparation of PCR-quality mouse genomic DNA with hot sodium hydroxide and tris (HotSHOT). *Biotechniques* 29 52–54. 10907076 10.2144/00291bm09

[B56] Vannucci S. J. Maher F. Simpson I. A. (1997). Glucose transporter proteins in brain: delivery of glucose to neurons and glia. *Glia* 21 2–21. 9298843 10.1002/(sici)1098-1136(199709)21:1<2::aid-glia2>3.0.co;2-c

[B57] Wang J. Ma H. Tong C. Zhang H. Lawlis G. B. Li Y. (2010). Overnutrition and maternal obesity in sheep pregnancy alter the JNK-IRS-1 signaling cascades and cardiac function in the fetal heart. *FASEB J.* 24 2066–2076. 10.1096/fj.09-142315 20110268 PMC2874473

[B58] Woods A. Dickerson K. Heath R. Hong S. P. Momcilovic M. Johnstone S. R. (2005). Ca2+/calmodulin-dependent protein kinase kinase-beta acts upstream of AMP-activated protein kinase in mammalian cells. *Cell Metab.* 2 21–33. 10.1016/j.cmet.2005.06.005 16054096

[B59] Yamashita H. Shao J. Ishizuka T. Klepcyk P. J. Muhlenkamp P. Qiao L. (2001). Leptin administration prevents spontaneous gestational diabetes in heterozygous Lepr(db/+) mice: effects on placental leptin and fetal growth. *Endocrinology* 142 2888–2897. 10.1210/endo.142.7.8227 11416008

[B60] Yamauchi T. Kamon J. Minokoshi Y. Ito Y. Waki H. Uchida S. (2002). Adiponectin stimulates glucose utilization and fatty-acid oxidation by activating AMP-activated protein kinase. *Nat. Med.* 8 1288–1295. 10.1038/nm788 12368907

[B61] Yang J. Kalogerou M. Gallacher J. Sampson J. R. Shen M. H. (2013). Renal tumours in a Tsc1+/– mouse model show epigenetic suppression of organic cation transporters Slc22a1, Slc22a2 and Slc22a3, and do not respond to metformin. *Eur. J. Cancer* 49 1479–1490. 10.1016/j.ejca.2012.10.027 23228442

[B62] Yao G. Zhang Y. Wang D. Yang R. Sang H. Han L. (2017). GDM-induced macrosomia is reversed by Cav-1 via AMPK-mediated fatty acid transport and GLUT1-mediated glucose transport in placenta. *PLoS One* 12:e0170490. 10.1371/journal.pone.0170490 28125642 PMC5268469

[B63] Zhang B. Tan L. Yu Y. Wang B. Chen Z. Han J. (2018). Placenta-specific drug delivery by trophoblast-targeted nanoparticles in mice. *Theranostics* 8 2765–2781. 10.7150/thno.22904 29774074 PMC5957008

[B64] Zhang Y. Shan P. Srivastava A. Li Z. Lee P. J. (2019). Endothelial stanniocalcin 1 maintains mitochondrial bioenergetics and prevents oxidant-induced lung injury via toll-like receptor 4. *Antioxid. Redox Signal.* 30 1775–1796. 10.1089/ars.2018.7514 30187766 PMC6479262

[B65] Zhong Y. Lee K. Deng Y. Ma Y. Chen Y. Li X. (2019). Arctigenin attenuates diabetic kidney disease through the activation of PP2A in podocytes. *Nat. Commun.* 10:4523. 10.1038/s41467-019-12433-w 31586053 PMC6778111

[B66] Zhou J. Bondy C. A. (1993). Placental glucose transporter gene expression and metabolism in the rat. *J. Clin. Invest.* 91 845–852. 10.1172/JCI116305 8450065 PMC288036

[B67] Zhou R. R. Li H. B. You Q. S. Rong R. You M. L. Xiong K. (2019). Silencing of GAS5 alleviates glaucoma in rat models by reducing retinal ganglion cell apoptosis. *Hum. Gene Ther.* 30 1505–1519. 10.1089/hum.2019.056 31608710

