# Supplementary material for: Gestational Diabetes Mellitus-Associated Hyperglycemia Impairs Glucose Transporter 3 Trafficking in Trophoblasts Through the Downregulation of AMP-Activated Protein Kinase
Source: Front Cell Dev Biol. 2021 Nov 2;9:722024. doi: 10.3389/fcell.2021.722024 (PMC8593042; doi:10.3389/fcell.2021.722024)
Supplement: Supplementary file 1 [file Data_Sheet_1.docx]

| **Component Name** | **Analyte Mass Range** | **Analyte Retention Time (min)** | **p-value** | **Normal-1** | **Normal-2** | **Normal-3** | **Normal-4** | **Normal-5** | **GDM-1** | **GDM-2** | **GDM-3** | **GDM-4** | **GDM-5** | **QC1** | **QC2** | **QC3** |
| --- | --- | --- | --- | --- | --- | --- | --- | --- | --- | --- | --- | --- | --- | --- | --- | --- |
| D-Glucose 6-phosphate | 259.0 / 97.0 | 10.07630231 | 0.046* | 21712139.27 | 31226293.97 | 21902360.62 | 44503392.23 | 17704013.5 | 16934611.68 | 17325662 | 14159061.41 | 18256477.82 | 11909731.6 | 15614828.89 | 14256662.24 | 12522941.06 |
| Beta-D-Fructose 6-phosphate | 259.1 / 97.0 | 9.36464762 | 0.0442* | 5960784.434 | 10810942.06 | 5981810.274 | 14001501.78 | 5650058.973 | 5178971.142 | 6236021.482 | 2697219.617 | 3827930.323 | 2815148.974 | 3418291.851 | 2816979.255 | 2072562.136 |
| Dihydroxyacetone phosphate | 169.1 / 97.0 | 8.380868789 | 0.0461* | 5459977.745 | 8428382.827 | 4914425.283 | 10594494.28 | 6590751.116 | 4898918.093 | 4006190.589 | 6043544.596 | 4497525.556 | 2461344.101 | 3439028.722 | 3160213.353 | 2865694.362 |
| Phosphoenolpyruvate | 167.1 / 79.0 | 9.433025389 | 0.1971 | 23113938.32 | 28218080.05 | 22196206.8 | 56190882.49 | 11111059.14 | 9480908.307 | 17923214.55 | 28675269.06 | 17848677.43 | 7727988.514 | 17187685.57 | 14100102.82 | 10416254.65 |
| Pyruvate | 87.1 / 43.1 | 1.54 | 0.0323* | 16683.075 | 12697.55 | 9692.175 | 19115.1 | 17966.45 | 7848.645 | 9455.985 | 14703.92 | 8604.925 | 5529.732126 | 7632.175 | 13373.65 | 10638.15 |
| Lactate | 89.2 / 43.0 | 2.346085182 | 0.4364 | 8050402.526 | 2441787.189 | 6213641.516 | 3046680.846 | 3123164.298 | 660658.3927 | 3584303.042 | 6855063.772 | 1641155.739 | 3886552.427 | 1716379.714 | 1776412.9 | 1714368.53 |
| ADP | 426.0 / 79.0 | 9.592502995 | 0.8194 | 4276955.279 | 823111.5874 | 4525769.303 | 5726618.061 | 2626334.202 | 1757847.45 | 4032377.546 | 6113960.035 | 1730359.37 | 2950373.851 | 1878042.55 | 1674560.957 | 1512559.905 |
| ATP | 506.0 / 159.0 | 10.84316466 | 0.8769 | 160777.6564 | 31555.64907 | 203599.8778 | 265976.5335 | 52110.34126 | 52956.1232 | 209230.7306 | 242427.585 | 31378.97518 | 129293.9251 | 85509.8345 | 71470.63906 | 62126.40417 |

**Supplementary Table 1. The list of identified glycolytic intermediates in placentas.**

**P< 0.05*, Normal group VS. GDM group

QC, quality control

**Supplementary Table 2. The list of the details of the standard curves.**

| No. | Component Name (standards) | Mass Info | Retention Time | Linear | R^2^ | Standard concentration gradient |
| --- | --- | --- | --- | --- | --- | --- |
| 1 | D-Glucose 6-phosphate | 259.0 / 97.0 | 8.173530628 | y = 0.24727 x - 0.31928 | 0.99918 | 0.1µM, 0.25µM, 0.5µM, 1µM, 2.5µM, 5µM, 10µM, 25µM, 50µM, 100µM, 250µM |
| 2 | Beta-D-Fructose 6-phosphate | 259.1 / 97.0 | 7.228246295 | y = 0.16221 x -0.00556 | 0.99984 | 0.1µM, 0.25µM, 0.5µM, 1µM, 2.5µM, 5µM, 10µM, 25µM, 50µM, 100µM, 250µM |
| 3 | Dihydroxyacetone phosphate | 169.1 / 97.0 | 6.159421462 | y = 0.15595 x - 0.46680 | 0.99974 | 0.1µM, 0.25µM, 0.5µM, 1µM, 2.5µM, 5µM, 10µM, 25µM, 50µM, 100µM, 250µM |
| 4 | phosphoenolpyuvate | 167.1 / 79.0 | 8.82 | y = 0.0662 x - 0.6317 | 0.9974 | 10µM, 25µM, 50µM, 100µM, 250µM, 500µM |
| 5 | pyruvate | 87.1 / 43.1 | 1.419615795 | y = 0.0003 x -0.0039 | 0.9969 | 5µM, 10µM, 25µM, 50µM, 100µM |
| 6 | lactate | 89.2 / 43.0 | 2.078771192 | y = 0.0033 x - 0.0208 | 0.9988 | 25µM, 50µM, 100µM, 250µM, 500µM, 1mM |
| 7 | ADP | 426.0 / 79.0 | 7.296759479 | y = 0.38325 x + 0.08279 | 0.99974 | 0.1µM, 0.25µM, 0.5µM, 1µM, 2.5µM, 5µM, 10µM, 25µM, 50µM, 100µM, 250µM |
| 8 | ATP | 506.0 / 159.0 | 10.41 | y = 0.0664 x - 2.4018 | 0.9928 | 25µM, 50µM, 100µM, 250µM, 500µM |

**Supplementary Table 3. Primer sequences for RT-qPCR.**

| Primers | Sequence（5’-3’） |
| --- | --- |
| AMPKα1，transcript variant 1-F | ggcccagaggtagatatatg |
| AMPKα1，transcript variant 1-R | gatccacctgcagcatatgt |
| AMPKα1，transcript variant 2-F  AMPKα1，transcript variant 2-R  AMPKα2-F  AMPKα2-R  GLUT1-F  GLUT1-R  GLUT3-F  GLUT3-R  β-actin-F  β-actin-R | ctctttcctgaggatccatc  gccaaaggatcctggtgatt cccttcctatgatgctaacg agaactcactggcttggttc  TCACTGTCGTGTCGCTGTTTG  GCTCAGATAGGACATCCAGGGTA  TCCCCTCCGCTGCTCACTATTT  ATCTCCATGACGCCGTCCTTTC  TGGCACCCAGCACAATGAA  CTAAGTCATAGTCCGCCTAGAAGCA |

**Supplementary Table 4. Clinical characteristics of the subjects.**

|  | Normal | GDM |
| --- | --- | --- |
| Subjects | n=30 | n=32 |
| Maternal age (years) | 29.27±3.27 | 30.91±4.73 |
| Fasting plasma glucose (mmol/L)  1-h plasma glucose (mmol/L)  2-h plasma glucose (mmol/L)  Pre-pregnancy BMI (kg/m^2^)  BMI before birth (kg/m^2^)  Parity  Gestational duration (weeks)  Blood pressure (mmHg)  Systolic  Diastolic  Neonatal birth weight (g)  Placental weight (g) | 4.49±0.20  7.21±1.16  5.07±0.66  21.32±3.00  28.25±3.62  1.07±0.25  39.43±0.73  113.20±6.46  65.87±5.42  3432.00±389.70  552.3±47.39 | 5.29±0.64^***^  10.13±1.65^***^  8.53±1.36^***^  22.55±3.19  29.05±3.33  1.41±0.50^**^  39.30±0.79  115.70±6.48  68.50±6.63  3682.00±501.60^*^  559.40±74.70 |

BMI, body mass index. The data are the mean±SD and Student's t test was used between Normal and GDM group; *^***^ P < 0.0001, ^**^ P < 0.005, and ^*^P < 0.05 versus normal pregnancy*.

**
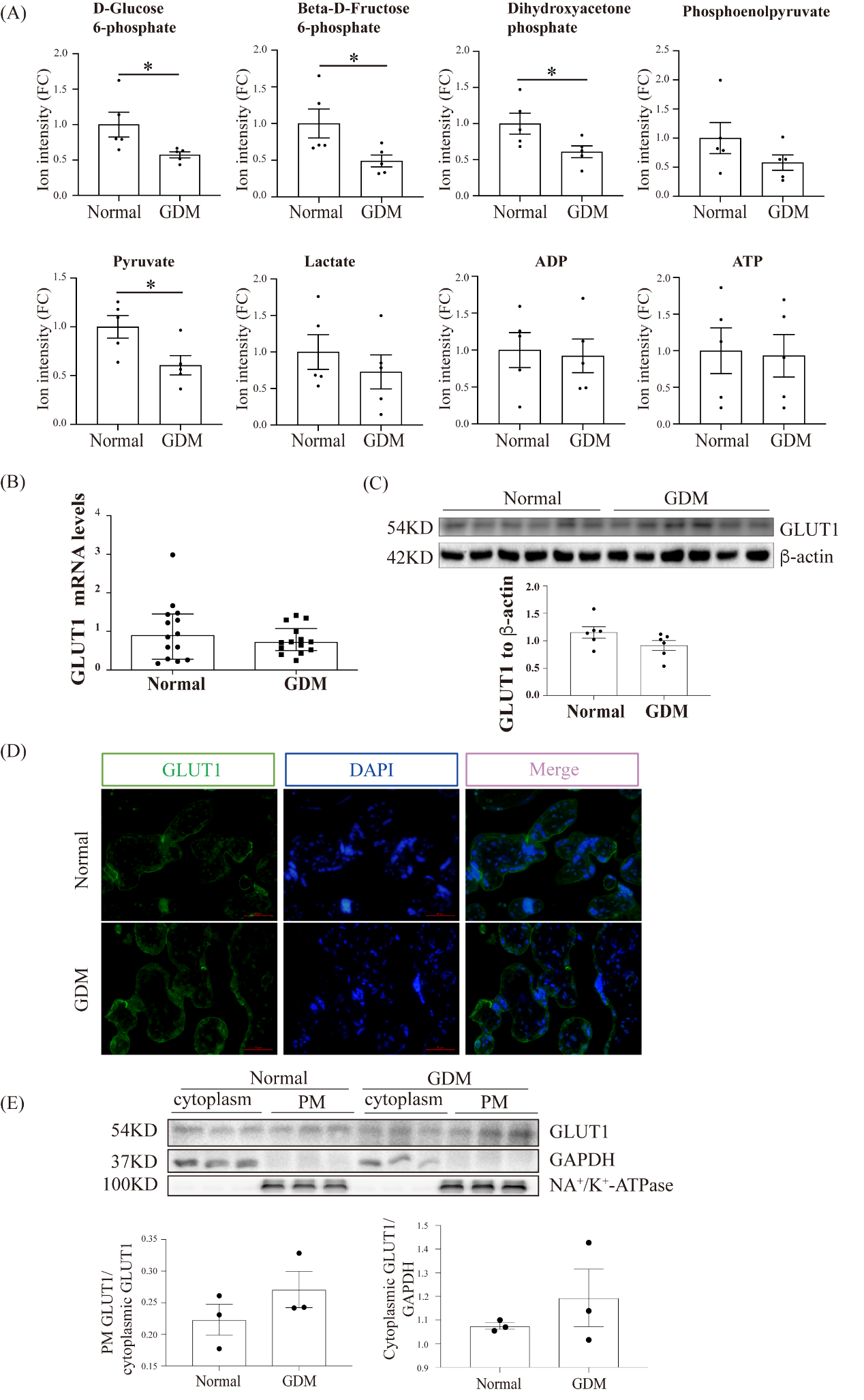
**

**Supplementary Figure 1**. GLUT1 expression in GDM-complicated human placentas.

**(A)** Graphs shows the glycolytic intermediates in normal and GDM-complicated placentas in consistent with the heatmap in Figure 1A. FC represents the ratio of the ion intensity of each compound of each sample to the average ion intensity of the compound of the normal group. n=5.

**(B)** mRNA levels of GLUT1 in GDM and control placentas, n=14; **(C)** GLUT1 protein levels in GDM and normal placentas, n=6; **(D)** IF staining of GLUT1 in normal and GDM-complicated human placentas. Nuclei were counterstained with DAPI. Bar: 50 μm; **(E)** plasma membranous and cytoplasmic GLUT1 protein levels in GDM and normal placentas, n=3. PM GLUT1: PM GLUT1 expression/ Na^+^/K^+^-ATPase expression; cytoplasmic GLUT1: cytoplasmic GLUT1 expression/actin expression. The data that are presented as the means ± SEMs are annotated in normal font, and Student's t test was used between two groups. The data presented as the medians (IQRs) are shown in bold font and Mann-Whitney test was used between two groups. **p < 0.05*, ***p < 0.01*, ****p < 0.001*.


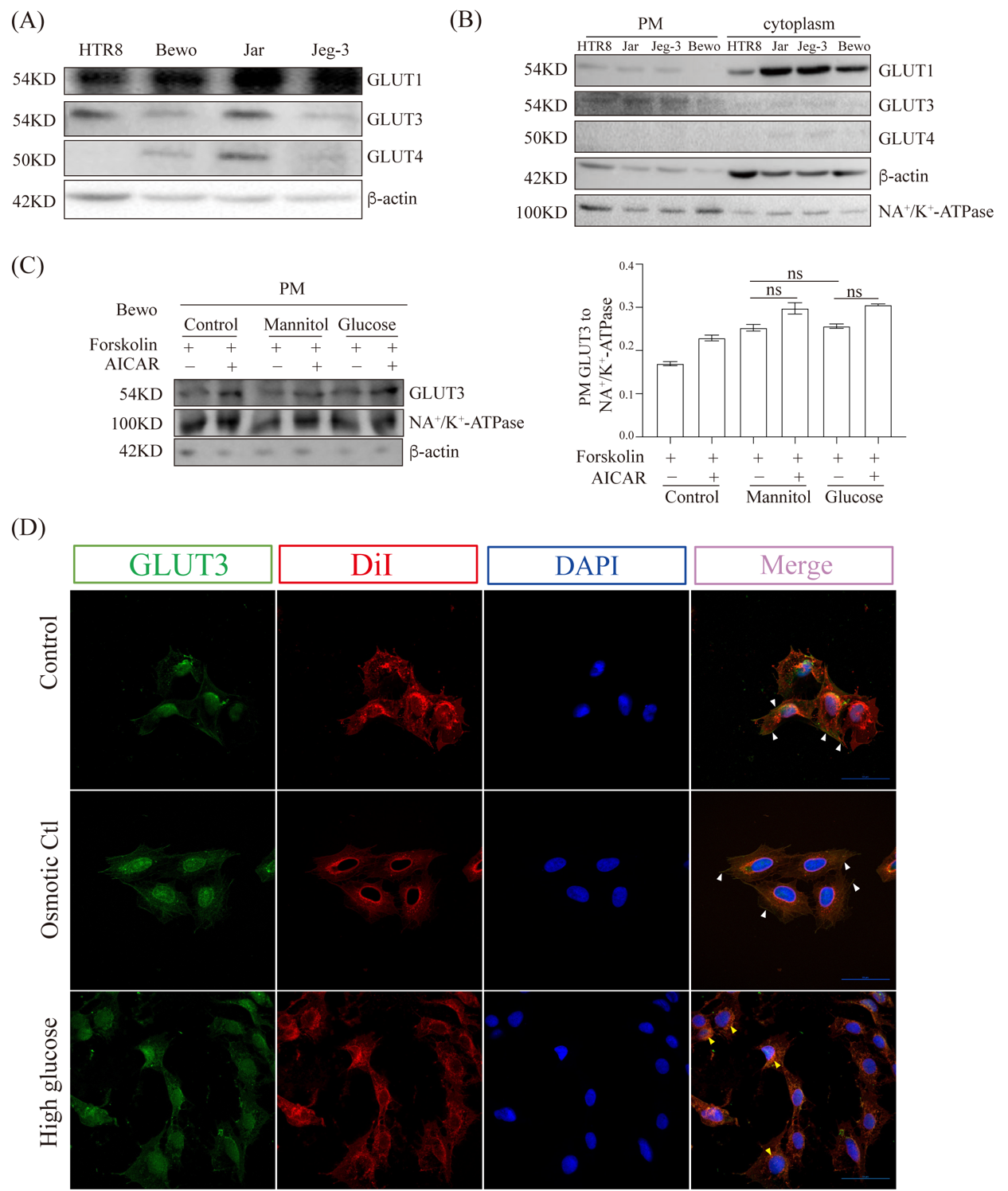


**Supplementary Figure 2**. Expression and distribution of GLUT3 in BeWo, Jar, Jeg-3 and HTR8/SVneo cells.

**(A)** protein expression levels of GLUT1, GLUT3 and GLUT4 in HTR-8/SVneo, Bewo, Jar, and Jeg-3 cells; **(B)** plasma membranous and cytoplasmic levels of GLUT1, GLUT3 and GLUT4 in HTR-8/SVneo, Bewo, Jar, and Jeg-3 cells;**(C)** syncytialization of Bewo cells was induced by 50μM forskolin for 24 h, and then the cells were incubated with fresh medium that containing 30 mM glucose (high glucose) , 7mM D-glucose +23 mM mannitol (osmotic control), or 7mM D-glucose (control), respectively. After 30 mimutes,10μM AICAR or vehicle was added for 24 h, GLUT3 protein in PM fraction was then determined by western blotting. PM GLUT3 was normalized to Na^+^/K^+^-ATPase. **(D)** IF staining of GLUT3 in HTR-8/SVneo cells using confocal microscopy. Plasma membrane were counterstained with DiI. Nuclei were counterstained with DAPI. White arrow：PM-GLUT3，Yellow arrow：intracellular membrane-GLUT3, Bar: 50 μm. The data presented as the medians (IQRs) are shown in bold font, and ordinary ANOVA test was used among multiple groups. **p < 0.05*, ***p < 0.01*, ****p < 0.001*.


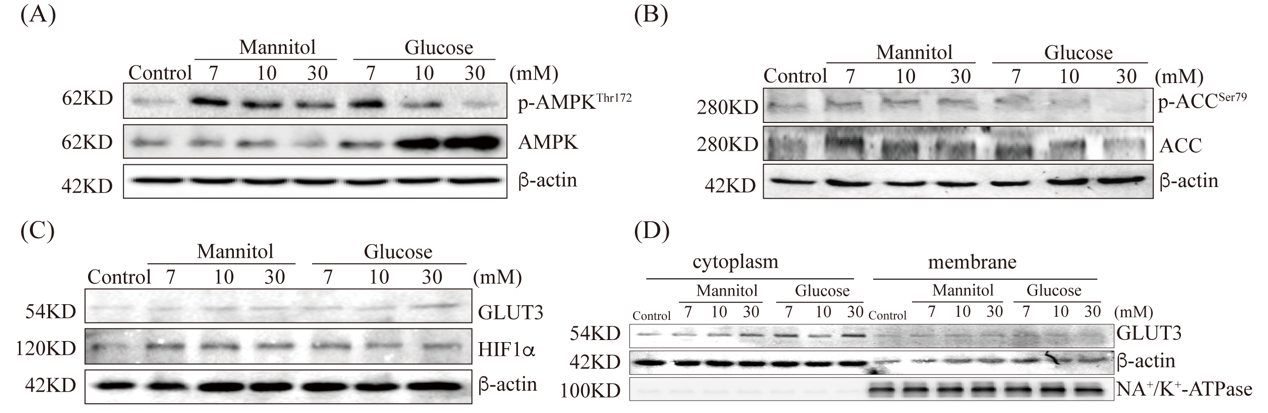


**Supplementary Figure 3**. AMPK-GLUT3 axis in HTR-8/SVneo cells in response to hyperglycemic stimuli.

HTR8/SVneo cells were treated with 7, 10, and 30 mM D-glucose respectively, while paralleled concentrations of mannitol were given as osmotic control, and a control group (11.1 mM D-glucose) was also included. After 24 h incubation, the cells were then subjected to western blotting of **(A)** p-AMPK and AMPK, **(B)** p-ACC and ACC; **(C)** GLUT3 and HIF-1α; **(D)** the levels of GLUT3 in membrane and cytoplasm fractions, respectively.


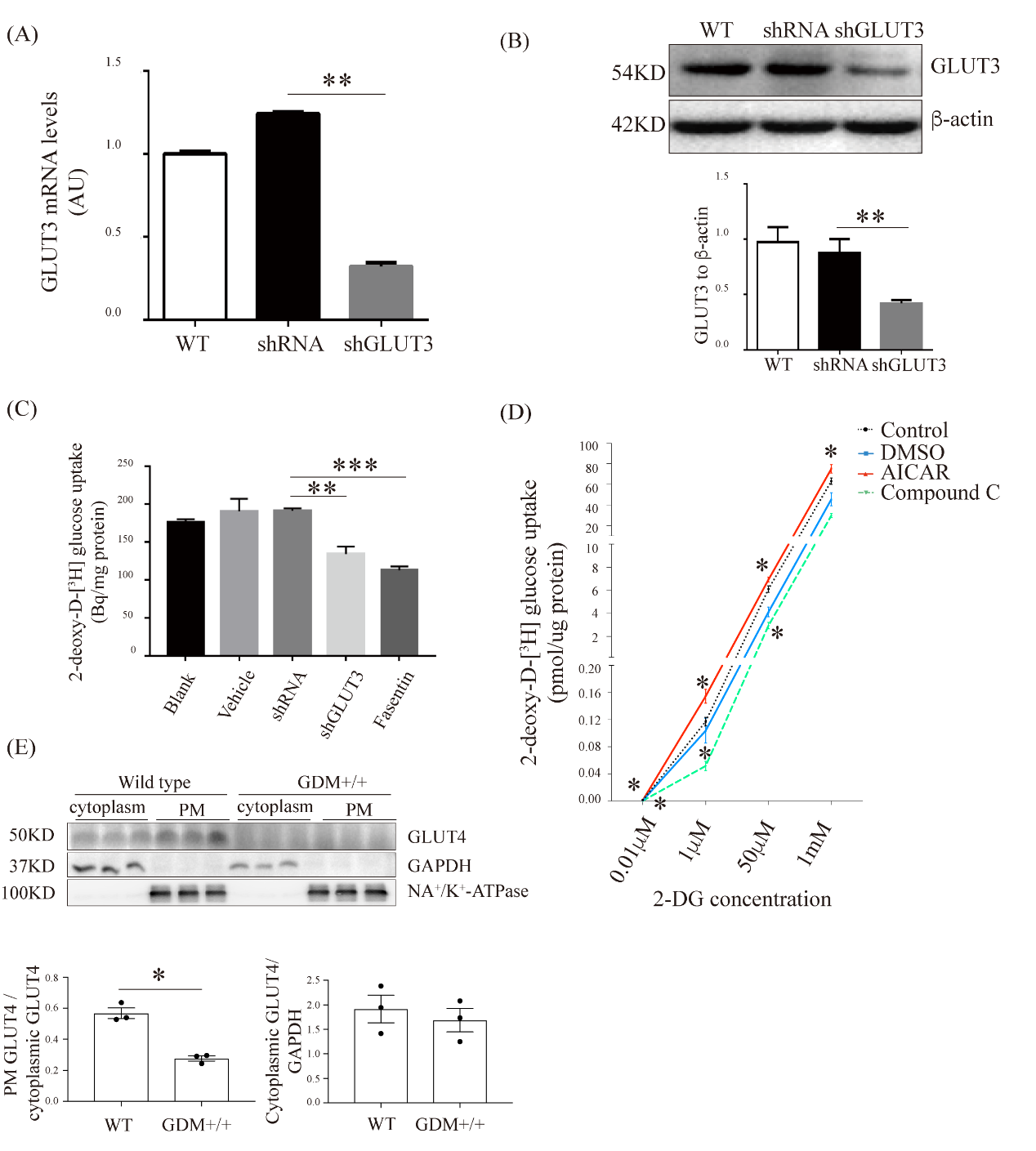


**Supplementary Figure 4**. Glucose uptake capacity of GLUTs in trophoblasts.

**(A)** mRNA levels of GLUT3 in wild-type (WT), shNC and shGLUT3-transfected HTR8/SVneo cells, n=3; **(B)** GLUT3 protein levels in WT, shNC and shGLUT3 HTR8/SVneo cells, n=3; **(C)** 2-deoxy-D-[^3^H] glucose uptake in HTR8/SVneo cells transfected with shGLUT3 or shNC or treated with vehicle (DMSO) or the GLUT1/4 inhibitor fasentin (100 µM), n=3; **(D)** 2-deoxy-D-[^3^H] glucose uptake in HTR8/SVneo cells pretreated with 200μM AICAR or 20μM Compound C or DMSO and then treated with different concentrations of 2-deoxy-D-glucose (0.01μM, 1μM, 50μM and 1mM) for 24h, n=3-4 per group; **(E)** plasma membranous and cytoplasmic GLUT4 protein levels in GDM-complicated and normal mouse placentas, n=6. The data that are presented as the means ± SEMs are annotated in normal font, and Student's t test was used between two groups while ordinary ANOVA test was used among multiple groups. The data presented as the medians (IQRs) are shown in bold font, and Mann-Whitney test was used between two groups while Kruskal-Wallis test was used among multiple groups. **p < 0.05*, ***p < 0.01*, ****p < 0.001*.
